# Genome-wide identification and characterization of NCED gene family in soybean (*Glycine max* L.) and their expression profiles in response to various abiotic stress treatments

**DOI:** 10.1371/journal.pone.0319952

**Published:** 2025-03-25

**Authors:** Md Shohel Ul Islam, Pollob Shing, Mahin Ahmed, Fatema Tuz Zohra, Amina Rownaq, Suronjeet Kumar Paul, Shaikh Mizanur Rahman, Md. Abdur Rauf Sarkar

**Affiliations:** 1 Laboratory of Functional Genomics and Proteomics, Department of Genetic Engineering and Biotechnology, Faculty of Biological Science and Technology, Jashore University of Science and Technology, Jashore, Bangladesh; 2 Department of Genetic Engineering and Biotechnology, Faculty of Biological Sciences, University of Rajshahi, Rajshahi, Bangladesh; 3 Institute of Biological Sciences, University of Rajshahi, Rajshahi, Bangladesh; Gauhati University, INDIA

## Abstract

The NCED (9-cis-epoxy carotenoid dioxygenase) enzyme regulates the biosynthesis of abscisic acid (ABA), which is responsible for plant growth, development, and response to various environmental challenges. However, no genome-wide identification, characterization, functional regulatory element analysis, and expression profiles in response to different abiotic stresses of the *NCED* gene family have yet to be investigated in an economically important legume plant species, soybean (*Glycine max* L.). Through comprehensive analysis, 16 *NCED* genes (named *GmNCED1* to *GmNCED16*) belonging to the RPE65 domain were identified in the soybean genome and found to be unequally distributed over 9 distinct chromosomes. The distinct intron-exon structures of *GmNCED* genes were categorized into six groups and shared a close relationship with the grapevine. Segmental gene duplication events and the purifying selection process were evident in *GmNCED* genes, according to evolutionary studies. *Cis*-acting regulatory element analysis revealed that *GmNCED* genes were largely associated with light response as well as stress response. ERF, MYB, bZIP, and LBD emerged as the major transcription factors in *GmNCED* genes. The protein-protein interactions demonstrated the close relationship between *GmNCED* and *Arabidopsis thaliana* proteins, while micro-RNA analysis revealed the involvement of *GmNCED* genes in plant growth and development as well as in the regulation of abiotic stress. The expression profiles of *GmNCED2*, *GmNCED11*, and *GmNCED12* provided evidence of their engagement in dehydration and sodium salt stress, whereas *GmNCED14* and *GmNCED15* were up-regulated in drought stress. Moreover, the up-regulation of *GmNCED13* and *GmNCED14* genes in heat tolerant germinated seed stages at high temperature delta region. More specifically, *GmNCED14* might be used as a novel candidate gene under drought stress, and influencing seed germination at high temperature. Overall, this study identified the crucial role of *GmNCED* in conferring resistance against abiotic stress such as dehydration, salt, and drought, and also uncovering the detailed regulatory mechanism of ABA biosynthesis during seed germination.

## 1. Introduction

Plants have developed a wide range of defense mechanisms and strategies to sustain, endure, and recover from environmental challenges such as water scarcity, saltiness, heat, cold stress, heavy metal toxicity, disease, and others. Among these strategies, abscisic acid (ABA) biosynthesis machinery is prerequisite for the plant growth and development including germination and seed dormancy, leaf senescence, the architecture of roots, regulation of stomata, and vegetable development and resistance to abiotic stress [1]. ABA can be synthesized in plants by direct or indirect pathways, with most higher plants using the indirect C40 pathway [2]. In the C40 pathway, zeaxanthin epoxidase (ZEP) enzyme converts zeaxanthin to violaxanthin and ultimately to neoxanthin. The conversion of violaxanthin and neoxanthin into xanthoxin is mediated by NCED enzyme [3]. Xanthoxin, the immediate precursor of ABA, is subsequently converted into abscisic aldehyde by the action of the short-chain alcohol dehydrogenase enzyme, ABA2. Ultimately, abscisic aldehyde is then oxidized by abscisic aldehyde oxidase to form abscisic acid [4,5].

*NCED* belongs to the *CCD* gene family, which typically contains the conserved domain REP65 [6,7]]. The maize *V14* gene, expressed in embryos and roots, was the first *NCED* gene to be discovered and cloned [3,8]. Members of the *NCED* gene family have been studied in various plant species, including cotton (*Gossypium hirsutum*) [5], *Arabidopsis* (*Arabidopsis thaliana*) [9], grape (*Vitis vinifera*) [10], avocado (*Persea americana*) [11], and rice (*Oryza sativa*) [12]. In *Arabidopsis*, *AtNCED6* is constitutively expressed in the endosperm and interacts with *AtNCED9* to regulate seed dormancy and germination by regulating ABA levels [9,13–15]. When the *NCED* gene is expressed in seed, elevated levels of ABA is biosynthesized resulting in the seed retaining dormancy until favorable conditions for germination occur [16]. Conversely, *AtNCED3* is primarily induced by water stress and regulates endogenous ABA levels in water-stressed environments [9,17,18]. In rice, *OsNCED3* and *OsNCED4* alter the plant and leaf morphology and enhance drought stress resistance [19–21]. Moreover, elevated levels of reactive oxygen species (ROS) signal drought stress [22]. Under this conditions, plants synthesize primary metabolites (PMs) and secondary metabolites (SMs) and exclusively store SMs products such as terpenes, phenolic compounds, glycine-betaine, and proline to counteract the drought [23]. Alternatively, the up-regulation of the *NCED* gene triggers the different signaling cascade pathways including mitogen-activated protein kinase (MAPK) to increase the production of ABA, thus boosting stomatal closure and minimizing water loss through transpiration [24–26]. This entire mechanism is regulated by various transcription factors (TFs) including MYB, NAC, ABF, DREB/CBF, MYC, ERF, and bZIP [27]. ABA regulation through *NCED* also influences root modulation and lateral root formation under such conditions [28].

Soybean (*Glycine max* L.), globally recognized oil crop species, belonging to the Fabaceae family, is important for both its economic and nutritional value. Soybean is an essential protein for both human and animal consumption, making its growth and productivity crucial for global food security [29]. However, abiotic factors such as drought, salt, and severe temperatures often severely impact on soybean cultivation [30]. In response to these challenges, plants activate complex stress-responsive ABA signaling-mediated pathways [31].

The objective of this study is to identify and annotate the *GmNCED* genes and unveil their involvement in ABA biosynthesis during the seed germination and in response to dehydration, sodium salt and drought stress. Comprehensive bioinformatics approaches were used to elucidate *GmNCEDs* physical and chemical properties, phylogenetic relationship, conserved domain, motifs, gene structural feature analysis, evolutionary relationships, chromosome mapping, gene duplication, subcellular localization, *cis*-acting regulatory elements, gene ontology, micro-RNA analysis along with performing RNA-seq analysis. Overall, this study will shed light on the mechanisms behind the functional diversity and the involvement of ABA biosynthesis during seed germination and stress responses aiding in the development of improved soybean cultivars in future breeding programs.

## 2. Methods and materials

### 2.1. Database searching and retrieval of GmNCED protein sequences

*A. thaliana* NCED DNA-binding domains were employed to extract *NCED* gene-encoding proteins in *G. max* from phytozome version 13 (https://phytozome-next.jgi.doe.gov/) using BLASTp (Protein-basic local alignment search tool) with an expected (E) threshold value of -1, comparison matrix (BLOSUM62), and other default parameters (S1 Data) [32]. SMART (Simple Modular Architecture Research Tool; http://smart.embl-heidelberg.de/) [33] and the NCBI CDD (Conserved Domain Database; https://www.ncbi.nlm.nih.gov/Structure/cdd/wrpsb.cgi) were used to analyze conserved domains with default parameters [34]. These proteins encoding typical RPE65 domains were renamed according to the order of their physical chromosomal positions.

### 2.2 Determination of physio-chemical properties of GmNCED

The ProtParam online program (http://web.expasy.org/protparam/) was employed to determine the number of amino acid (a.a) residues, aliphatic index, molecular weight, instability index, isoelectric point (pI), and grand average of hydropathicity (GRAVY) of GmNCED proteins [35].

### 2.3. Phylogenetic relationship analysis between PpNCED, RcNCED, AtNCED, OsNCED, VvNCED, and GmNCED

NCED proteins derived from rose (*Rosa chinensis*) [36], grape vine (*Vitis* vinifera) [10], *Arabidopsis* (*Arabidopsis thaliana*) [9], peach (*Prunus persica*) [37], rice (*Oryza sativa*) [12], and soybean (*Glycine max*) were used to construct a phylogenetic tree (S2 Data). The MEGA11 software and ClustalW program were utilized to align the amino acid sequences and build the phylogenetic tree [38,39]. The NJ (neighbor-joining) method was used with 1000 bootstrap value and keeping other as default parameters. The constructed tree was uploaded to iTOL online tool version 6.7.4 (https://itol.embl.de/) for attractive visualization [40].

### 2.4. Gene structure analysis of *GmNCED*

CDS sequence (S3 Data) and genomic sequence (S4 Data) were uploaded to the Gene Structure Display Server database version 2.0 (GSDSv2.0; http://gsds.cbi.pku.edu.cn/) to analyze the *GmNCED* genes structure [41].

### 2.5. Conserved domain and motif analysis of GmNCED

InterPro database (http://www.ebi.ac.uk/interpro/) was employed to predict the conserved domains and visualized using TBtools software version 1.116 [42]. The structural motif of the GmNCED proteins was analyzed in Multiple EM for Motif Elicitation (MEME) tools of MEME-suite (https://meme-suite.org/meme/), selecting a maximum number of motifs 20 with other default parameters [43]. The motifs were visualized using the MEME online interface, employing the MEME and motif scanning approach.

### 2.6. Evolutionary divergence time and Ka/Ks ratio calculation in *GmNCED*

The *GmNCED* gene family Ka (non-synonymous) and Ks (synonymous) substitution ratio was computed using the Ka/Ks calculator (https://bio.tools/kaks_calculator) [44]. The duplication and time of divergence measured in million years ago (MYA) of *GmNCED* genes were determined using the formula T = Ks/2λ, where λ is equal to 6.5 × 10^-9^ [45]. The data was converted into log2 format in a heat map in TBtools to visualize the evolutionary relationship and divergence rate.

### 2.7. Collinearity and synteny analysis of *GmNCED*

Collinear and synteny analysis were performed based on the gene duplication events in *GmNCED* genes. Subsequently, the syntenic pairs with *A. thaliana*, *O. sativa,* and *V. vinifera* and collinear relationship within *GmNCED* genes were visualized in TBtools.

### 2.8. Chromosomal mapping and duplication analysis of *GmNCED*

The distribution of *GmNCED* genes across the chromosomes was mapped and visualized in the MapGene2Chrom online tool version 2.0 (MG2C; http://mg2c.iask.in/mg2c_v2.0/) [46]. The duplication of *GmNCED* genes was also illustrated.

### 2.9. Subcellular localization of *GmNCED*

The Wolf PSORT online tool (https://wolfpsort.hgc.jp/) was utilized to determine the subcellular localization of *GmNCED* genes [47]. The predicted protein signals were illustrated employing RStudio software version 2023.06.1 [48].

### 2.10. *Cis*-acting regulatory elements (CAREs) analysis of *GmNCED*

The 2000 bp from 5′ untranslated region (5′ UTR) was used to predict CAREs (http://bioinformatics.psb.ugent.be/webtools/plantcare/html/) (S5 Data) [49]. The predicted CAREs were classified and visualized in heatmap using TBtools.

### 2.11. Gene ontology (GO) analysis of *GmNCED*

GO analysis was performed by extracting GO IDs from Plant Transcriptional Regulatory Map database (PlantRegMap; https://plantregmap.gao-lab.org/go.php) with *p*-value 0.01 and other default parameters [50]. Additionally, GO enrichment was visualized in chiplot online tool (https://www.chiplot.online/) [51].

### 2.12. Transcription factors (TFs) analysis of *GmNCED*

TFs binding site prediction in *GmNCED* genes was performed in the Plant Transcriptional Regulatory Map database (PlantRegMap; https://plantregmap.gao-lab.org/binding_site_prediction.php) using threshold *p*-value 1.0E-4 and other default parameter.

### 2.13. Regulatory network between TFs and *GmNCED*s

Cytoscape software version 3.9.1 was utilized to construct and display the interaction network between TFs and predicted *GmNCED* genes [52].

### 2.14. Prediction of putative micro-RNAs (miRNAs) and network targeting *GmNCED*

The sequences of the putative miRNA of *GmNCED* were extracted from miRBase (https://mirbase.org/) [53]. The CDS sequences of *GmNCED* were uploaded to psRNATarget Server18 (https://www.zhaolab.org/psRNATarget/analysis?function=2) that target *GmNCED* genes keeping other parameters as default [54]. Furthermore, the interaction network of the predicted miRNAs and *GmNCED*s was illustrated in cytoscape.

### 2.15. Protein-protein interaction (PPI) prediction of GmNCED

*A. thaliana* homologous proteins were used to predict the PPI network of GmNCED proteins in the string online tool version 12 (https://string-db.org/) [55]. The parameters of the string were kept as network type-full STRING network; the meaning of network edges-evidence; active interaction source-text mining, experiments, databases, co‑expression, neighborhood, gene fusion, co‑occurrence; minimum required interaction score-medium confidence parameter (0.4); maximum number of interactions display 1^st^ shell-no more than 10, with 2^nd^ shell was left blank; and enabling network display options as 3D bubble design.

### 2.16. Transcriptomic data analysis of *GmNCED* under salt, dehydration, and drought stresses

The RNA-seq data for salt and dehydration (GSE57252), drought stress (GSE69469) was extracted from the NCBI’s Gene Expression Omnibus (GEO; https://www.ncbi.nlm.nih.gov/geo/) [56]. A heat map was generated in TBtools to illustrate the expression pattern of *GmNCED*. The normalized FPKM (Fragments Per Kilobase per Million mapped fragments) value was log2 transformed to distinguish expression levels in the particular gene.

### 2.17. Transcriptomic data analysis of *GmNCED* during various seed developmental stages

The RNA-seq data included with two soybean lines; PI 587982A, a heat-tolerant landrace, and S99-11986, a conventional high-yielding adapted line) during three seed germination stages: (1) 6 h imbibed seed; (2) germinated seed; and (3) dry and mature seed, which were produced in two regions, considering the low-temperature south region, and heat stressed field located in the delta region [57]. The Sequence Read Archive (SRA) under bio-project PRJNA509794 was obtained from NCBI to perform the RNA-seq analysis. For trimming and quality control of the RNA-seq, trimmomatic package version 0.32 was used [58]. Subsequently, star package version 2.7.11b was utilized to align the RNA sequencing with the reference genome *G. max* [59]. Samtools package version 1.20 converted sequence alignment map (SAM) files to binary alignment map (BAM) files [60]. RSEM package version 1.1.17 was used to calculate the FPKM value [60,61]. Chiplot was used to visualize the expression pattern of *GmNCED* genes.

## 3. Results

### 3.1. Physicochemical characteristics of GmNCED

The physicochemical properties of GmNCED proteins were analyzed, revealing variation from 239 (GmNCED13) to 618 a.a (GmNCED12), with a mean length of 508.87 a.a (Table 1). The molecular weights of the encoded proteins estimated to range from 26559.57 (GmNCED13) to 69373.56 kDa (GmNCED12). The pI measurement indicated that 11 GmNCEDs had pI value less than 7.0, classifying them as acidic, while five GmNCEDs had pI value greater than 7.0, indicating an alkaline nature. According to the instability index analysis, there were five GmNCED proteins (GmNCED5, GmNCED8, GmNCED9, GmNCED11,GmNCED16) that had instability index value exceeding 40.0. Whereas, there were eleven GmNCED proteins (GmNCED1, GmNCED2, GmNCED3, GmNCED4, GmNCED6, GmNCED7, GmNCED10, GmNCED12, GmNCED13, GmNCED14,GmNCED15) that had instability index value lower than 40.0. The aliphatic index varied, with GmNCED7 contained the highest aliphatic of 84.70 and GmNCED10 and GmNCED11 possessing the lowest aliphatic index of 75.16. All the GmNCED proteins were found to be hydrophilic as indicated by their negative GRAVY score.

**Table 1:** List of 16 GmNCEDs and their basic physiochemical characterizations.

Gene name	Gene identifier	Size (a.a)	Mass (kDa)	pI	Instability index	Aliphatic index	GRAVY
*GmNCED1*	Glyma.01G073200	614	69313.18	7.99	37.71	78.58	−0.389
*GmNCED2*	Glyma.01G154900	590	64979.48	6.42	39.20	81.12	−0.219
*GmNCED3*	Glyma.04G083500	579	64966.27	5.78	35.16	84.35	−0.206
*GmNCED4*	Glyma.04G083600	282	31819.61	7.03	38.28	76.03	−0.325
*GmNCED5*	Glyma.04G084100	566	63351.02	6.35	40.55	79.08	−0.363
*GmNCED6*	Glyma.05G140900	588	65605.72	6.01	39.17	77.28	−0.297
*GmNCED7*	Glyma.06G085000	581	65404.60	5.40	32.85	84.70	−0.193
*GmNCED8*	Glyma.06G085100	587	66599.27	6.48	45.05	82.49	−0.243
*GmNCED9*	Glyma.06G085800	563	62979.67	6.22	42.22	80.87	−0.321
*GmNCED10*	Glyma.08G096200	589	65933.24	6.72	38.84	75.16	−0.343
*GmNCED11*	Glyma.08G176300	611	67842.16	7.70	41.62	75.16	−0.398
*GmNCED12*	Glyma.11G161947	618	69373.56	9.00	38.17	77.77	−0.351
*GmNCED13*	Glyma.12G236650	239	26559.57	8.43	29.46	77.41	−0.306
*GmNCED14*	Glyma.12G236700	287	32648.14	5.53	34.99	79.79	−0.365
*GmNCED15*	Glyma.13G202200	543	60938.68	6.33	30.57	79.30	−0.315
*GmNCED16*	Glyma.15G250100	305	34450.33	5.48	40.75	81.11	−0.363

### 3.2. Phylogenetic relationship analysis between PpNCED, RcNCED, AtNCED, OsNCED, VvNCED, and GmNCED

**Fig 1:**
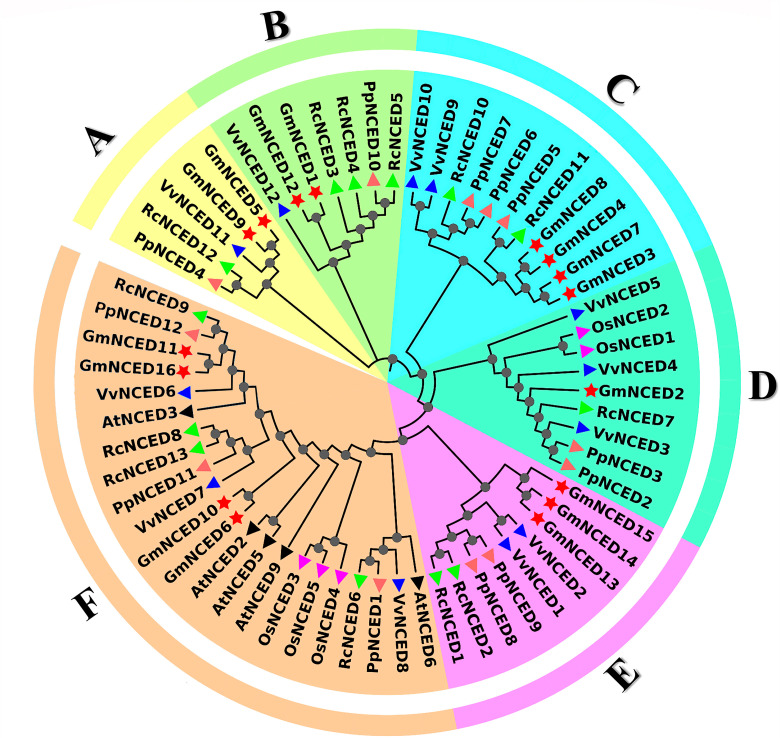
Phylogenetic relationship between GmNCED and RcNCED, PpNCED, VvNCED, AtNCED, and OsNCED. GmNCED was classified into 6 groups (A, B, C, D, E, and F), each marked by different colors and shapes. The red color star labeled the GmNCED. Whereas, RcNCED was labeled as triangular green, PpNCED was labeled as the light carmine pink triangle, VvNCED was labeled dark blue triangular, AtNCED was labeled as black triangular, and OsNCED was labeled as the magenta color triangle.

A phylogenetic tree was constructed using the full-length a.a sequence between *P. persica* (12 PpNCEDs) *R. chinensis* (13 RcNCEDs), *A. thaliana* (5 AtNCEDs), *O. sativa* (5 OsNCEDs), *V. vinifera* (12 VvNCEDs), and *G. max* (16 GmNCEDs) (Fig 1). The phylogenetic tree categorized 63 NCED proteins into six distinct groups; A, B, C, D, E, and F. Group F consisted with the highest overall NCED protein count (22), while group A had the least number (5). Among GmNCED proteins distribution among groups, group C emerged with the highest number (4). Meanwhile, group D contained the least number (1) (S6 Data). But, no AtNCED or OsNCED proteins were found in group A, B, C, and E.

### 3.3. Gene structure analysis of *GmNCED*

**Fig 2:**
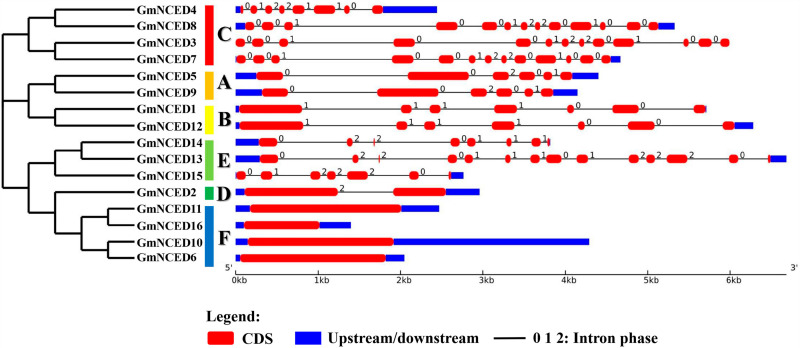
The gene structure of *GmNCED* genes. The grouping and colors of the *GmNCED* gene family members are based on the phylogenetic relationship. For the color bar, red represents exons, deep blue lines represent upstream/downstream and 0, 1, 2 represents intron phase.

The structural diversity of *GmNCED* genes were analyzed by comparing the distribution patterns of intron and exons. *GmNCED*s genes exhibited a range of 0 to 13 introns and the highest intron count (43) observed in group C (Fig 2; S7 Data). *GmNCED13,* a member of group E, contained maximum number of introns (13). Additionally, no introns were found in group F. *GmNCED*s genes exhibited a range of 1 to 14 exons, with group C consisting the largest number of exons (47). *GmNCED13*, from group E, contained the highest number of exons (14). The least number of exons were found in groups F, where *GmNCED6*, *GmNCED10*, *GmNCED11*, and *GmNCED16* contained one exon each.

### 3.4. Conserved domain and motif analysis of GmNCED

**Fig 3:**
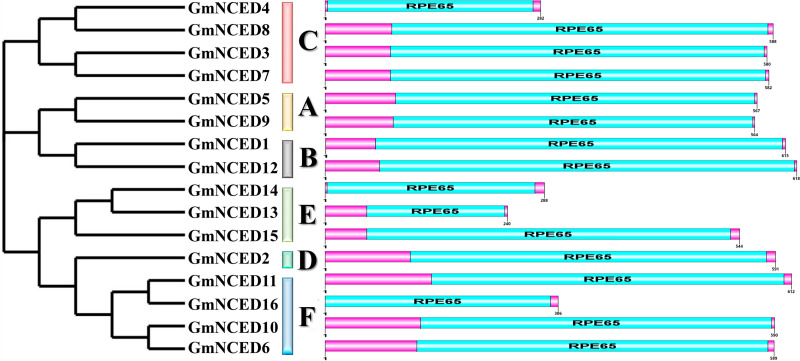
Feature domains of GmNCED proteins. The positions of the RPE65 conserved domain are demonstrated in sky blue color whereas the entire protein sequence of respective GmNCED is magenta colored.

**Fig 4:**
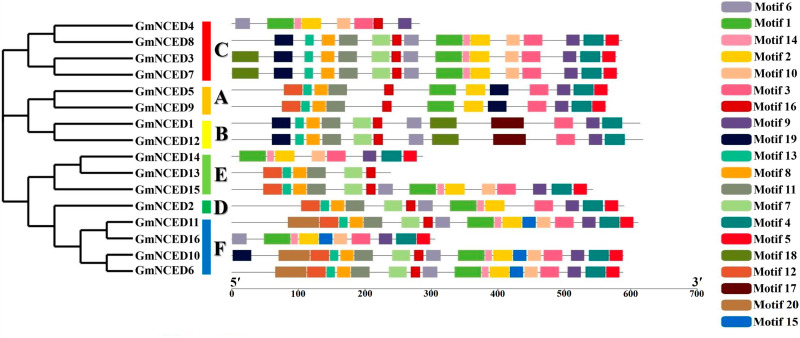
The distribution of conserved motifs in GmNCED proteins. Each motif is illustrated by a specific-colored box aligned on the right side of the figure. Different colors indicate individual motifs identified within each protein domain.

Conserved domain analysis revealed that all GmNCED proteins possessed an RPE65 domain (Fig 3). A total of 20 different conserved motifs were analyzed in the GmNCED proteins (Fig 4). All GmNCEDs, except for GmNCED13, possessed more than seven motifs. In contrast, GmNCED13 consisted of six motifs. Both GmNCED5 and GmNCED9 in group A shared the same motif patterns. Similarly, GmNCED1 and GmNCED12 in group B contained 12 motifs sharing similar motif structure. Further, motif 17 was only found in GmNCED1 and GmNCED12. The motifs varied among GmNCED14, GmNCED13, and GmNCED15 in group E and between GmNCED11 and GmNCED16 in group G. GmNCED10 contained the highest number of motifs (18), sharing all but motif 19 with GmNCED6. Motif 3 and Motif 9 were shared by all GmNCEDs except for GmNCED13. Additionally, all the motif logos varied in structure (S1 Fig).

### 3.5. Evolutionary divergence time and Ka/Ks ratio calculation in *GmNCED*

**Fig 5:**
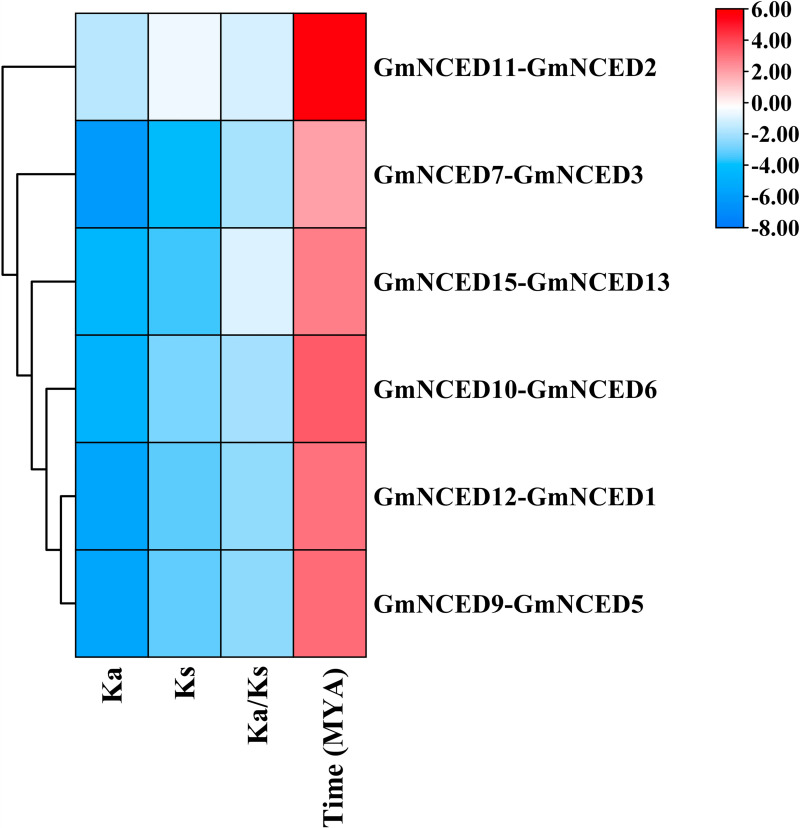
The estimation of divergence time and Ka/Ks ratio of *GmNCED* genes. The ratio of nonsynonymous (Ka) and synonymous (Ks) is represented by Ka/Ks. The time of divergence (measured in million years ago, MYA) is also represented. The different color bar represents the data range.

The Ka value of *GmNCED* gene pairs varied between 0.01406467 and 0.31955, while the Ks value ranged from 0.05235362 to 0.671 (Fig 5). The ratio of Ka/Ks was within the range of 0.188673269 to 0.51972207. This indicated that they evolved primarily under purifying selection (S8 Data). The duplication time of the *GmNCED* gene pairs ranged from 3.990367378 to 51.14329268 MYA, with *GmNCED11*-*GmNCED2* gene pairs showing evolutionary origin of 51.14329268 MYA.

### 3.6. Collinear relationship analysis of *GmNCED*

**Fig 6:**
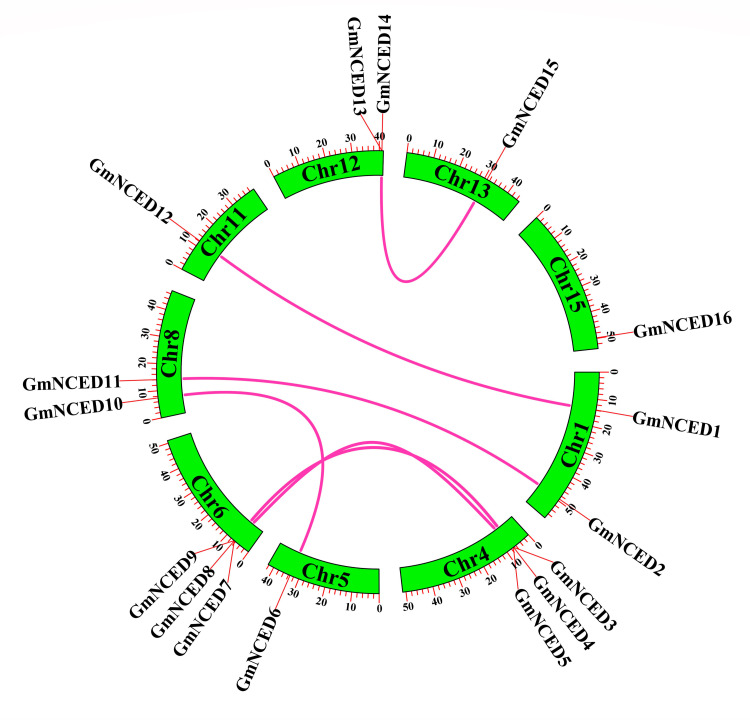
The collinearity analysis of the *GmNCED* gene family. Green color rectangles represent chromosomes of *GmNCED*. The purple red-colored lines linked between chromosomes represent collinear relations between the chromosomes.

The results indicated that single *GmNCED* gene was found on chromosome 5, 11, 13, and 15, while more than one *GmNCED* genes were found on chromosome 4 and 6 (Fig 6). In addition, *GmNCED12* had a collinear gene pair with *GmNCED1*, *GmNCED11* with *GmNCED2*, *GmNCED3* with *GmNCED7*, *GmNCED9* with *GmNCED5*, *GmNCED6* with *GmNCED10*, and *GmNCED15* with *GmNCED13*, resulting in a total of 6 pairs. Yet, *GmNCED16*, located on chromosome 15, did not have any collinear gene pairs.

### 3.7. Syntenic relationship analysis of *GmNCED*

**Fig 7:**
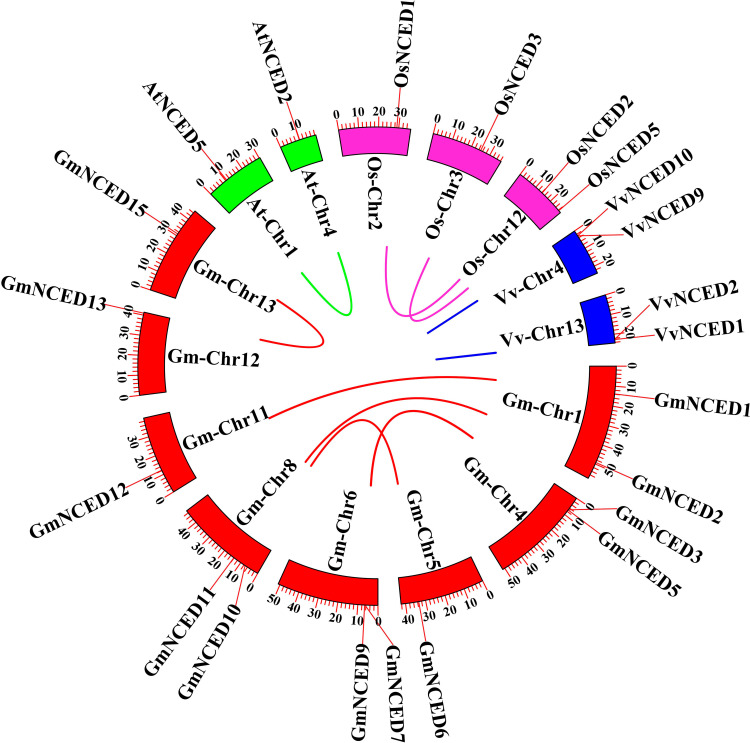
The synteny analysis between *GmNCED*, *AtNCED*, *OsNCED*, *VvNCED* genes chromosome. Red color rectangles represent the *GmNCED* chromosomes. Meanwhile, green rectangles represent *AtNCED* chromosomes. Furthermore, magenta color rectangles represent *OsNCED* chromosomes while blue color rectangles represent *VvNCED* chromosomes. The same color format is used to represent the syntenic relationship linkage between different species.

The syntenic map was constructed between *G. max* (*GmNCED*) and of three other different plant species, two dicotyledonous notably *V. vinifera* (*VvNCED*) and *A. thaliana* (*AtNCED*), as well as the monocotyledonous *O. sativa* (*OsNCED*) (Fig 7). Unfortunately, no syntenic gene pairs were observed among *GmNCED* genes.

### 3.8. Chromosome mapping and duplications analysis of *GmNCED*

**Fig 8:**
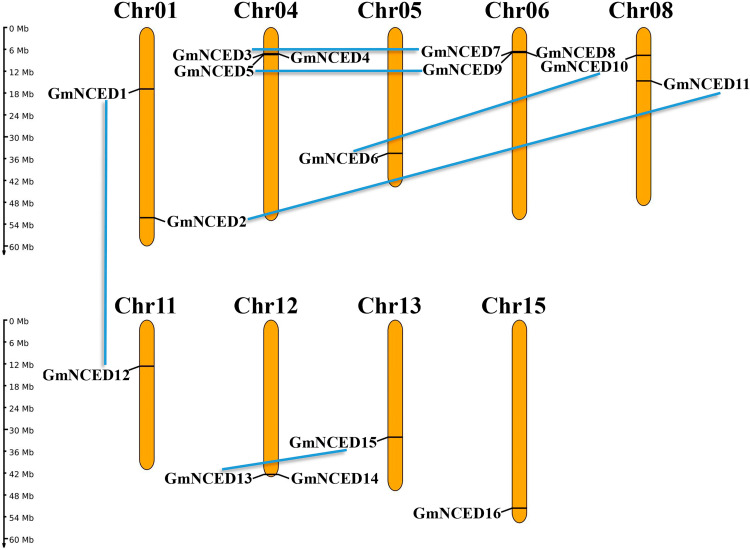
The chromosomal mapping and duplications of *GmNCED* genes. The number of distinct chromosomes is at the top of each chromosome bar. The chromosome scale is in millions of bases (Mb), indicating the length of each chromosome on the left. The chromosome is colored yellow, while sky blue lines indicate segmental duplications.

The analysis of chromosomal localization revealed that *GmNCED* genes were unequally distributed across the 9 different chromosomes (Fig 8). Chromosome 4 and 6 contained the highest number of *GmNCED* genes (3 each). Chromosome 4 (*GmNCED4*), chromosome 6 (*GmNCED8*), chromosome 12 (*GmNCED14*), and chromosome 15 (*GmNCED16*) were the independent chromosomes. Six segmental duplicated gene pairs; *GmNCED11*-*GmNCED2*, *GmNCED10*-*GmNCED6*, *GmNCED15*-*GmNCED13*, *GmNCED12*-*GmNCED1*, *GmNCED9*-*GmNCED5*, and *GmNCED7*-*GmNCED3* were identified. Nevertheless, no evidence of tandem duplication was seen in the *GmNCED* gene family.

### 3.9. Subcellular localization analysis of *GmNCED*

**Fig 9:**
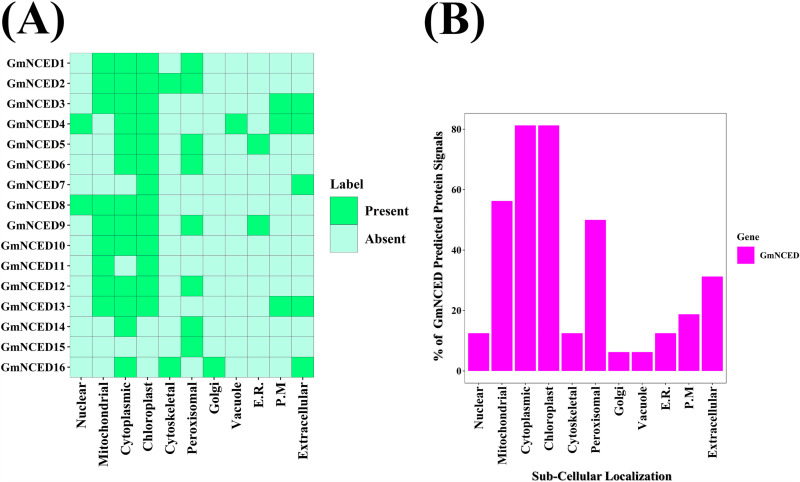
Sub-cellular localization analysis of *GmNCED* genes. **A. The heatmap represents the sub-cellular localization analysis of *GmNCED* genes.** The names of each *GmNCED* gene are shown on the left side of the heatmap, while the names of the respective cellular organelles are shown at the bottom of the heatmap. The intensity of color on the right side of the heatmap indicates the presence of protein signals corresponding to the genes. **B. The percentage distribution of *GmNCED* gene signal across various cellular organelles is represented by a bar diagram.** The percentages of protein signals appearing in different cellular organelles are shown on the left side of the diagram.

The subcellular localization analysis revealed that the most of the *GmNCED*s were located in the cytoplasm and chloroplast, with 13 *GmNCED* genes found in these organelles (Fig 9A). *GmNCED4* was observed in highest number of organelles (6). Golgi apparatus and vacuole possessed one *GmNCED* each, which is *GmNCED16*, and *GmNCED4* respectively. Additionally, fewer number of *GmNCED* genes were conserved in the nucleus, cytoskeletal, endoplasmic reticulum, and plasma membrane. Moreover, a significant percentage of *GmNCED* genes were also found in mitochondria, peroxisomal, and extracellular regions (Fig 9B). Moreover, the specific number of *GmNCED* genes that were found in a particular organelle was illustrated using a bubble plot (S2 Fig).

### 3.10. *Cis*-acting regulatory elements (CAREs) analysis of *GmNCED*

**Fig 10:**
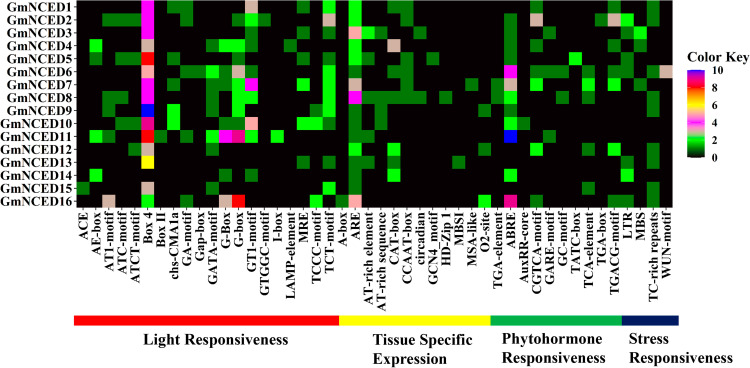
The distribution of putative *cis*-acting regulatory elements on the 2.0 kb promoter region of *GmNCED* is represented by a heatmap. The names of each *GmNCED* are shown on the left side of the heatmap. The number of putative *cis*-acting elements for each *GmNCED* gene is displayed on the right side of the heatmap and is represented by distinct colors. Functions associated with *cis*-acting elements of the corresponding genes, such as light responsiveness, tissue-specific expression, phytohormone responsiveness, and stress responsiveness, are shown at the bottom of the heatmap and labeled as red, yellow, green, and dark blue respectively.

46 CAREs were identified and classified into four distinct categories according to their functional regulation: light responsiveness, tissue-specific expression, phytohormone responsiveness, and stress responsiveness (Fig 10; S9 Data). Among these 46 CAREs, light responsiveness was the biggest group which included 20 elements such as GA-motif, Gap-box, GT1-motif, ACE, G-Box, G-box, AE-box, AT1-motif, ATCT-motif, Box 4, Box II, chs-CMA1a, GATA-motif, GTGGC-motif, I-box, LAMP-element, TCT-motif, MRE and TCCC-motif. Box 4 exhibited the highest number of *cis* elements in this category. The second-largest group was phytohormone responsiveness, which was composed of methyl jasmonate (MeJA) response elements (CGTCA-motif and TGACG-motif), salicylic acid (SA) response elements (TCA-element), gibberellin (GA) response elements (GARE-motif and TATC-box), abscisic acid (ABA) response elements (ABRE), anoxic specific inducibility (GC-motif), and auxin-responsive elements (TGA-element, AuxRR-core, and TGA-box). ABA response element (ABRE) was the largest group of phytohormone responsiveness-related *cis-*elements. The third largest group was tissue-specific expression which included A-box, ARE (anaerobic induction), AT-rich element (DNA binding protein), AT-rich sequence (maximal elicitor-mediated activation), CAT-box (meristem expression), CCAAT-box (MYBHv1 binding site), circadian (circadian control), GCN4_motif (endosperm expression), HD-Zip 1 (differentiation of the palisade mesophyll cells), MBSI (flavonoid biosynthetic gene regulation), MSA-like (cell cycle regulation), and O2-site (zein metabolism regulation). The most significant group was stress responsiveness, which included LTR (low-temperature responsive elements), TC-rich repeats (defense and stress-responsive elements), MYB (MYB binding sites involved in drought inducibility), and WUN-motif (wound-responsive elements).

### 3.11. Gene Ontology (GO) analysis of *GmNCED*

**Fig 11:**
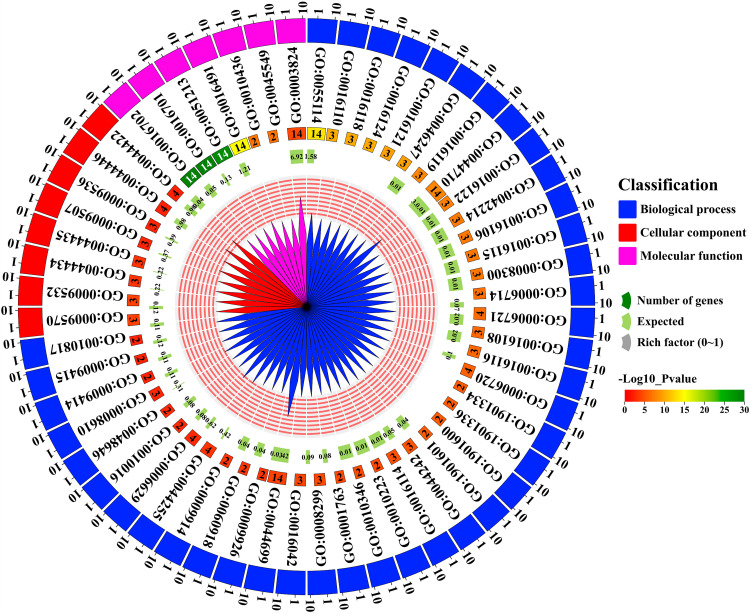
*GmNCED* gene’s function analysis through gene ontology. Classification of the *GmNCED* genes function are shown in circos plot. The number of genes involved under a certain GO ID, expected value, and rich factor are shown in a distinctive color. The scaling of the -log10 *p*-value is shown in three distinctive colors (red, yellow, and green).

56 GO IDs were identified in *GmNCED* genes and classified into three categories according to their respective functions; biological process (BP), cellular component (CC), and molecular function (MF) (Fig 11; S10 Data). The biological process was found predominantly among the three categories, which comprised 41 GO IDs; GO:0055114 (*p*-value: 5.20E-14), GO:0016110 (p-value:1.10E-10), GO:0016118(*p*-value: 1.10E-10), GO:0016124 (*p*-value: 1.10E-10), GO:0016121(*p*-value: 4.40E-10), GO:0046247 (*p*-value: 4.40E-10), GO:0016119 (*p*-value: 1.30E-08), GO:0044710 (*p*-value: 1.70E-08), GO:0016122 (*p*-value: 3.10E-08), GO:0042214 (*p*-value: 9.00E-08), GO:0016106 (*p*-value: 1.10E-07), GO:0016115 (*p*-value: 1.10E-07), GO:0008300 (*p*-value: 1.30E-07), GO:0006714 (*p*-value: 3.60E-07), GO:0006721 (*p*-value: 6.10E-07), GO:0016108 (*p*-value: 7.80E-07), GO:0016116 (*p*-value: 7.80E-07), GO:0006720 (*p*-value: 2.30E-06), GO:1901334 (*p*-value: 5.20E-06), GO:1901336 (*p*-value: 5.20E-06), GO:1901600 (*p*-value: 5.20E-06), GO:1901601 (*p*-value: 5.20E-06), GO:0044242 (*p*-value: 1.10E-05) GO:0016114 (*p*-value: 1.50E-05), GO:0010223 (*p*-value: 3.80E-05) GO:0010346 (*p*-value: 3.80E-05), GO:0001763 (*p*-value: 4.20E-05), GO:0008299 (*p*-value: 5.30E-05), GO:0016042 (*p*-value: 7.90E-05), GO:0044699 (*p*-value: 0.00014), GO:0009926 (*p*-value: 0.00048), GO:0060918 (*p*-value: 0.00061), GO:0009914 (*p*-value: 0.00066), GO:0044255 (*p*-value: 0.00066), GO:0006629 (*p*-value: 0.00265), GO:0010016 (*p*-value: 0.00266), GO:0048646 (*p*-value: 0.00298), GO:0008610 (*p*-value: 0.00333), GO:0009414 (*p*-value: 0.00495), GO:0009415 (*p*-value: 0.00518), GO:0010817 (*p*-value: 0.00613). In addition, the cellular component included 8 GO functions; GO:0009570 (*p*-value: 0.00011), GO:0009532 (*p*-value: 0.00012), GO:0044434 (*p*-value: 0.00078), GO:0044435 (*p*-value: 0.00082), GO:0009507 (*p*-value: 0.00364), GO:0009536 (*p*-value: 0.00425), GO:0044446 (*p*-value: 0.00617), GO:0044422 (*p*-value: 0.00625) and the molecular component included 7 GO functions; GO:0016702 (*p*-value: 1.00E-30), GO:0016701 (*p*-value: 1.00E-30), GO:0051213 (*p*-value: 2.30E-29), GO:0016491 (*p*-value: 1.30E-15), GO:0010436 (*p*-value: 1.70E-07), GO:0045549 (*p*-value: 1.70E-07), GO:0003824 (*p*-value: 5.20E-05)

Furthermore, the *p*-value in the BP, CC, and MF function categories were analyzed. Within the BP function category, GO:0010817 had the highest *p*-value of 0.00613, followed by GO:0009415 with a *p*-value of 0.00518, and then GO:0009414 with a *p*-value of 0.00495, and so on. Among the GO terms in the CC function category, GO:0044422 had the highest *p*-value of 0.00625, followed by GO:0044446 with a *p*-value of 0.00617, and subsequently GO:0044434 with a *p*-value of 0.00425. In the MF function category, GO:0003824 had the highest *p*-value of 0.000052, followed by GO:0010436 with a *p*-value of 0.00000017, and subsequently GO:0045549 with a *p*-value of 0.00000017. In BP, the single-organism process (GO:0044699, *p*-value: 0.00014), oxidation-reduction process (GO:0055114, *p*-value: 5.20E-14), and the single-organism metabolic process (GO:0044710, *p*-value: 1.70E-08) were predominantly seen. There were also a lot of different types of activities in the MF category. A few notable activities included oxidoreductase activity, acting on single donors and adding molecular oxygen (GO:0016702, *p*-value: 1.00E-30), oxidoreductase activity, which acts on single donors and adds molecular oxygen (GO:0016702, *p*-value: 1.00E-30), dioxygenase activity (GO:0051213, *p*-value: 2.30E-29), catalytic activity (GO:0003824, *p*-value: 5.20E-05) and oxidoreductase activity (GO:0016491, *p*-value: 1.30E-15).

### 3.12. Transcription factors (TFs) analysis of *GmNCED*

**Fig 12:**
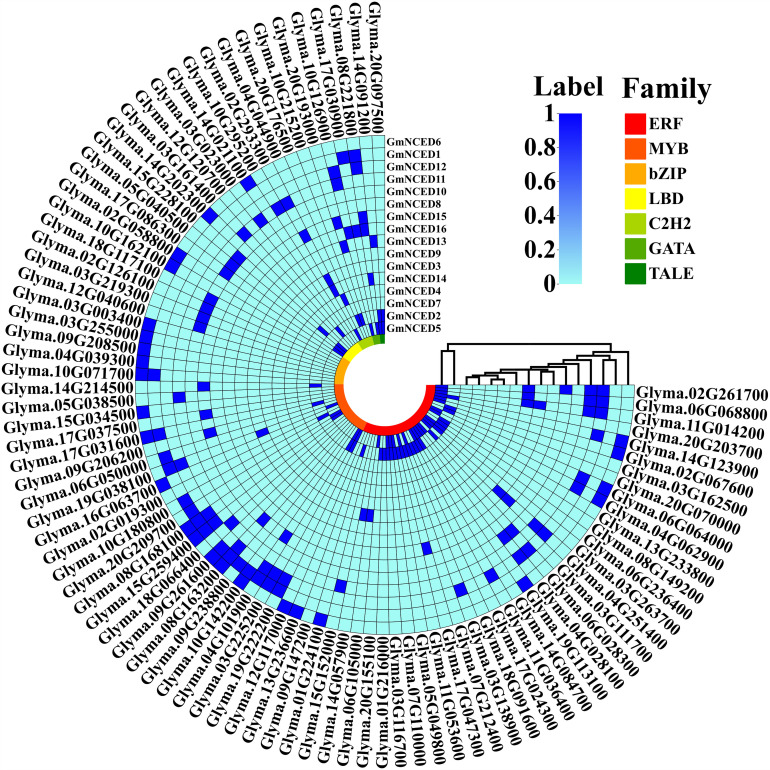
A heatmap represents transcription factors (TFs) in *GmNCED* genes. The color intensity of the heatmap indicates the presence of TFs corresponding to the proteins. The TFs are distributed into 7 TF families recognized by distinctive color. The 7 TFs family are ERF, MYB, bZIP, LBD, C2H2, GATA, and TALE which are colored red, light green, light red, orange, yellow, lime, light green, and dark green respectively.

In this analysis, an overall 91 unique TFs were found which regulate the 16 *GmNCED* genes. The identified TFs were categorized into seven distinct families, including ERF, MYB, bZIP, LBD, C2H2, GATA, and TALE (Fig 12). Among them, four major families such as ERF, MYB, bZIP, and LBD included 39, 22, 11, and 8 TFs respectively accounting for 87.91% of overall 91 detected TFs.

### 3.13. Regulatory relationship between TFs and *GmNCED*

**Fig 13:**
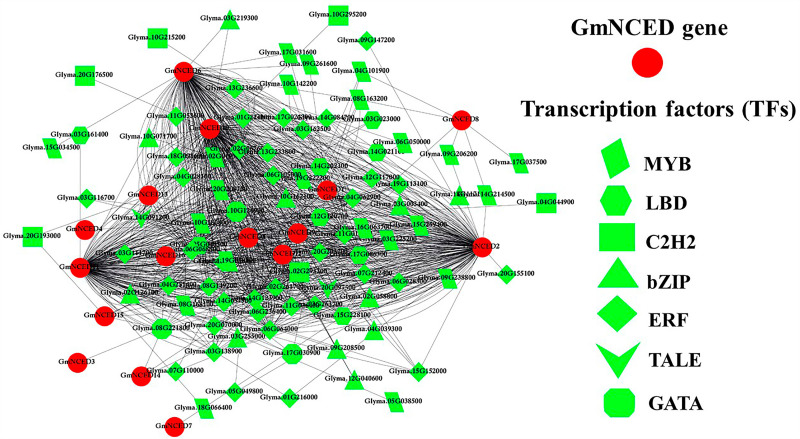
The regulatory network between TFs and *GmNCED* genes. The *GmNCED* is shown in round red. Whereas, the TFs are shown in green colors and different shapes. The 7 TF families ERF, MYB, C2H2, GATA, bZIP, LBD, and TALE have represented the shapes as diamond, parallelogram, rectangular, octagonal, triangle, hexagonal, and V respectively.

The sub-network connection between TFs and *GmNCED* genes were predicted (Fig 13). The sub-network analysis findings indicated that ERF was associated with eleven *GmNCED* genes except *GmNCED1*, *GmNCED3*, *GmNCED4*, *GmNCED7*, and *GmNCED14*. In addition, MYB TF family was associated with *GmNCED1*, *GmNCED2*, *GmNCED5*, *GmNCED6*, *GmNCED8*, *GmNCED9*, *GmNCED10*, *GmNCED11*, and *GmNCED12*. Similarly, LBD TF family constructed the regulatory relationship with *GmNCED2*, *GmNCED5*, *GmNCED6*, *GmNCED7*, *GmNCED9*, *GmNCED10* and *GmNCED11*. Furthermore, GATA and TALE TF family linked to *GmNCED1*, *GmNCED2*, *GmNCED5*, *GmNCED6*, *GmNCED9*, *GmNCED10*, *GmNCED11*, *GmNCED12*, *GmNCED14*, *GmNCED15*, *GmNCED16* and *GmNCED2*, *GmNCED5*, *GmNCED6*, *GmNCED9*, *GmNCED10*, *GmNCED11*, *GmNCED13* respectively. The C2H2 TF were associated with *GmNCED2*, *GmNCED3*, *GmNCED4*, *GmNCED5*, *GmNCED6*, *GmNCED10*, *GmNCED11*, *GmNCED13*, *GmNCED14* and *GmNCED15*. However, there were only four *GmNCED* genes (*GmNCED1*, *GmNCED6*, *GmNCED10*, and *GmNCED11*) in the bZIP family.

### 3.14. Prediction of putative micro-RNAs (miRNAs) and network targeting *GmNCED*

**Fig 14:**
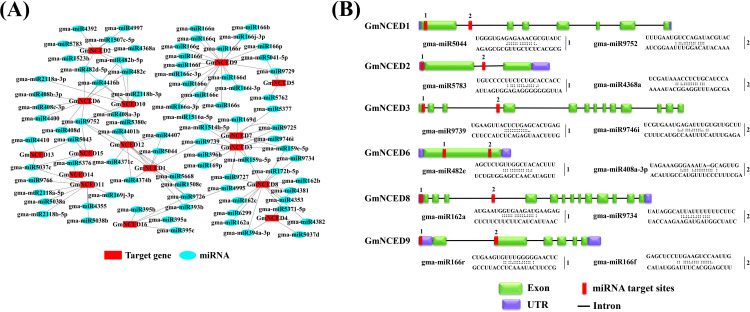
Prediction of potential micro-RNAs targeting *GmNCED* genes. **A.** Network illustration of predicted miRNA targets *GmNCED* genes. The red rectangle represents *GmNCED* genes while microRNA is labeled as sky blue ellipse. **B.** The schematic diagram indicates the *GmNCED* genes targeted by miRNAs. The green round rectangular is shown as exons of the respective gene, the blue round rectangular represents UTR, the straight black line represents intron and the red color small round rectangular represents miRNA.

In this analysis, 126 mature miRNAs targeting all 16 genes of *GmNCED* were shown in the network illustration (Fig 14A; S11 Data) and the schematic diagrams indicate the *GmNCED* genes targeted by miRNAs (Fig 14B). The data analysis further showed that about 53 unique miRNA sequences were present. It was identified that 16 members of gma-miR166 targeted one gene in particular *GmNCED9* (Table 2). Moreover, 9 members of gma-miR482 targeted *GmNCED2*, *GmNCED6*, and *GmNCED10*. Whereas, 6 family members of gma-miR159 targeted *GmNCED3*, *GmNCED7* and *GmNCED8* on the contrary 5 members of gma-miR9752 targeted 5 *GmNCED* genes (*GmNCED1*, *GmNCED6*, *GmNCED10*, *GmNCED12* and *GmNCED16*). However, the majority of the miRNA targeted two or more genes for instance 3 members of gma-miR169 targeted *GmNCED3* and *GmNCED11*. In the context of genes, GmNCED9 was targeted the most 19 times on the contrary, *GmNCED8* was targeted 15 times. Whereas, *GmNCED1*, *GmNCED3*, and *GmNCED6* were observed to be targeted up to 10 times.

**Table 2:** Information about abundant miRNA ID, functions, and their targeted *GmNCED* genes.

miRNA ID	Functions	Targeted genes
gma-miR166	It regulates gibberellic acid metabolism. It plays crucial role in plant growth, development and seed germination stages.	*GmNCED9*
gma-miR482	It plays a role in abiotic stress such as drought and salt. It also fights against pathogens.	*GmNCED2*, *GmNCED6*, *GmNCED10*
gma-miR9752	It plays a role in nodule formation.	*GmNCED1*, *GmNCED6*, *GmNCED10*, *GmNCED12*, *GmNCED16*

### 3.15. Protein-protein interaction (PPI) prediction of GmNCED

**Fig 15:**
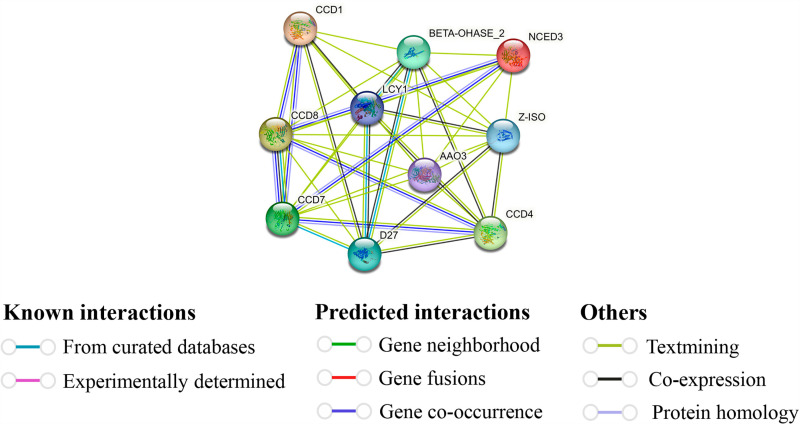
Protein-protein interaction of GmNCED proteins based on known *Arabidopsis* proteins. The proteins were displayed at network nodes with the proteins in nodes, and the line colors indicate different data sources.

GmNCED was identified as string protein based on its higher homology with *Arabidopsis*. The result showed that 15 GmNCED proteins interacted with *Arabidopsis* proteins (S12 Data). Six GmNCED proteins (GmNCED3, GmNCED7, GmNCED8, GmNCED13, GmNCED14 and GmNCED15) were observed to homologous with AtCCD1 (Fig 15). Furthermore, AtCCD1 interacted with CCD7, CCD8, D27, LCY1, Z-ISO and BETA-OHASE2. On the contrary, GmNCED6, GmNCED10, GmNCED11, and GmNCED16 were observed to carry the same feature as AtNCED3. AtNCED3 linked with LCY1, CCD7, AAO3, Z-ISO and BETA-OHASE2. AtCCD7 and AtCCD8 were noticed to be homologous with two GmNCEDs GmNCED1, GmNCED12, and GmNCED5, GmNCED9 respectively. They both interacted with CCD1, CCD4, AA03, NCED3, LCY1, and Z-ISO. However, AtCCD4 was only homologous with GmNCED2.

### 3.16. Transcriptomic analysis of *GmNCED* in dehydration, salt, and drought stress

**Fig 16:**
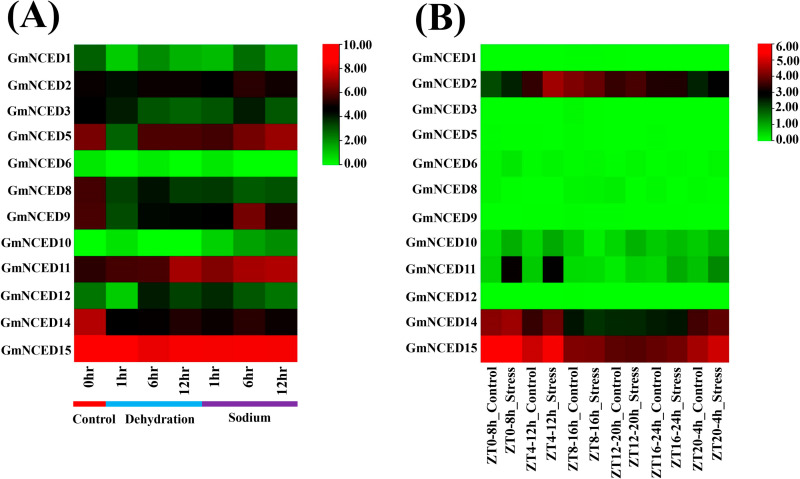
Transcriptomic profiling of *GmNCED* in dehydration, salt, and drought stress. **A.** The name of the respective *GmNCED* genes are shown on the left side of the heatmap. The bottom of the heatmap contains the control, dehydration, and sodium salt treatment at different hours. **B.** The name of the respective *GmNCED* genes are shown on the left side of the heatmap. The bottom of the heatmap contains the control and drought stress treatment at different hours. The FPKM value are transformed into the log2 format and are shown in the color gradient from low to high expression (green to red color) on the right side of both the heatmaps.

The RNA-seq data showed the expression pattern of *GmNCED* genes under dehydration, salt, and drought stress. The expression analysis of the *GmNCED* genes under dehydration treatment (1hr, 3hr, and 6hr) showed that the most of the *GmNCED* genes were down-regulated compared to the control (Fig 16A; S13 Data). The expression of *GmNCED15* was observed higher than any other gene though it had showed down-regulation. One gene in particular, *GmNCED11*, showed up-regulation of its expression in 1hr, 3hr, and 6hr water stress. Two genes (*GmNCED6* and *GmNCED10*) showed little or no expression both in the control and treatment states. Under salt stress, the expression pattern in some instances showed similarity with water stress, however, the expression under salt stress showed some differences at 1hr, 3hr, and 6hr of sodium treatment. The expression of *GmNCED2* at 6hr and 12hr sodium treatment showed the up-regulation of expression. *GmNCED5* was observed to be up-regulated at 12hr though at 3hr and 6hr, the expression was down-regulated. *GmNCED9* was observed to express higher than the control whereas *GmNCED11* showed up-regulation of its expression in 1hr, 3hr, and 6hr sodium treatment revealing its ability to be expressed in salt conditions. Under drought stress, the expression of *GmNCED* genes in the drought treatment group divided into 4hr time intervals from lights came on (8:00 a.m. =  Zeitgeber Time (ZT) 0), during a 24hr time course (ZT0, ZT4, ZT8, ZT12, ZT16, and ZT20) (Fig 16B; S14 Data). Under drought stress, the expression of most genes was observed insignificant as no real differences were monitored whether it was control or treatment group. However, some notable genes such as *GmNCED2*, *GmNCED11*, *GmNCED14*, and *GmNCED15* showed up-regulation in the expression at different time intervals upon drought treatment.

### 3.17. Transcriptomic analysis of *GmNCED* during seed developmental stages

**Fig 17:**
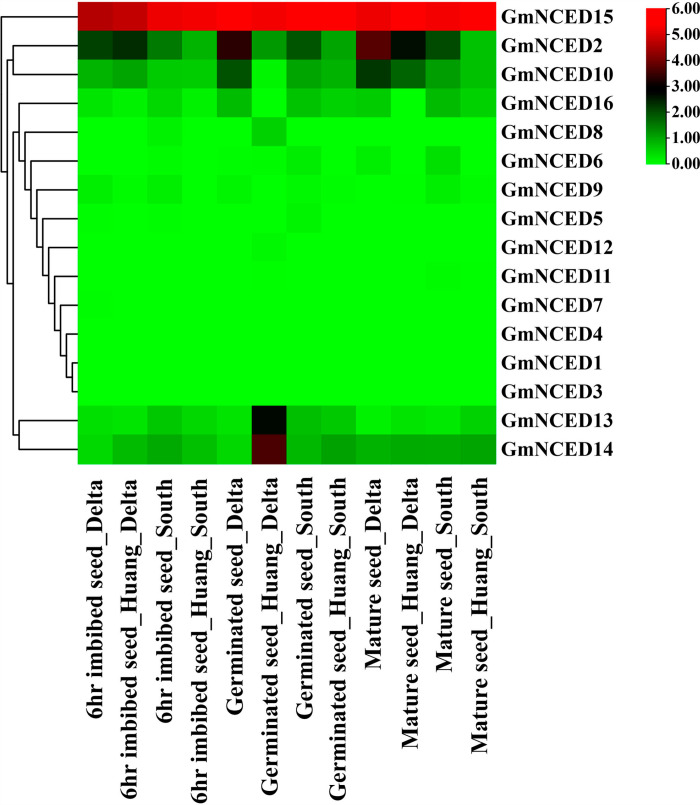
Transcriptomic profiling of *GmNCED* genes in seed developmental stages in the south and delta region. The name of the respective *GmNCED* genes are shown on the left side of the figure. The bottom of the heatmap contains different stages of high yielding and heat tolerant seed; 6hr imbibed seed, germinated seed, and mature seed produced in south and delta region. The color gradient (white to red color), on the right side of the heatmap, shows low to high expression.

The results from RNA-seq provided that in high temperature delta region, approximately 44% *GmNCED* genes expressed in high-yielding adapted 6hr imbibed seed where *GmNCED2* and *GmNCED15* expressed highly (Fig 17; S15). In contrast, 38% *GmNCED* genes expressed in heat-tolerant 6-hr imbibed Huang seed, with *GmNCED2, GmNCED10* and *GmNCED15* up-regulated in that region. When 6hr imbibed seed was produced in south region, 50% *GmNCED* genes expressed whereas heat-tolerant Huang showed expression of *GmNCED* genes approximately 38%. Meanwhile, *GmNCED15* was the single up-regulated genes and rest of the *GmNCED* genes were down-regulated. When germinated seeds were cultivated in delta region, about 38% *GmNCED* genes expressed. However, the rate of expression was decreased to 31% when Huang was cultivated in same region with the up-regulation of *GmNCED8, GmNCED13* and *GmNCED14.* In the southern region, expression rate of *GmNCED* gene in two types germinated seed were nearly equivalent. *GmNCED14* and *GmNCED15* exhibited up regulation in contrast *GmNCED2, GmNCED6 GmNCED10, GmNCED13* and *GmNCED16* exhibited down-regulation. The number of expressed *GmNCED* genes for mature seed of high-yielding and Huang cultivated in delta region were seven and five respectively. *GmNCED13, GmNCED14* and *GmNCED15* considered as up-regulated expressed genes. In the southern region, mature seed of high yielding and Huang expressed approximately 50% and 38% respectively where three up-regulated genes (*GmNCED13, GmNCED14* and *GmNCED15)* and five down-regulated genes *(GmNCED2, GmNCED6, GmNCED9, GmNCED10 and GmNCED16*) were observed.

## 4. Discussion

*NCED* genes are involved in the biosynthesis of ABA mediated stress response. Therefore, it is indispensable to be acquainted with the physiochemical properties of GmNCED proteins. The instability index indicated their overall stability, with values consistently below 40.0. Additionally, variations in the aliphatic index implied differences in their structural properties. In addition, all GmNCED proteins were hydrophilic. *Rosaceae* species revealed a relatively similar evolutionary relationship and demonstrated the expansion of the *NCED* gene family during the evolution of the *Prunus* genus [37]. An in-depth knowledge of a species evolutionary history is crucial step comprehending the evolutionary relationships of genes. In the phylogenetic tree, the proximity of genes within a cluster directly correlates with the similarity of their functions [62]. Most of the GmNCEDs were clustered with VvNCEDs, indicating GmNCEDs were closely related to the VvNCEDs. Additionally, the absence of *AtNCED* and *OsNCED* genes in multiple groups might indicate a divergence in the genetic composition of the species.

The seven groups varied in the intron-exon structure and number. The presence of many introns in *GmNCED15* allows for the possibility of alternative splicing, indicating specific biological functions [63]. On the contrary, the absence of introns in group F indicated a simpler gene structure. It was found genes with longer introns have showed elevated expression [64]. *GmNCED6*, *GmNCED10*, *GmNCED11*, and *GmNCED16* categorized as early response genes and activated more quickly because of fewer exons [65,66].

Moreover, all the GmNCED protein possessed RPE65 domain which is essential for the enzymatic oxidation of carotenoids [6]. Thus, GmNCED demonstrated a similar distribution pattern across different groups. *GmNCED* genes motif varied across different groups and were relatively similar in the same group. *PbNCED* genes motif arrangements were identical within the same subgroup but varied among different subgroups [67]. Moreover, the absence of motif 17 in *GmNCED13* highlighted the distinct characteristics within group E. *GmNCED5* and *GmNCED9* in group A shared the same motif indicating that they might work together in related biological processes.

Ka/Ks ratio calculation highlighted that the duplicated *PbNCED* gene pairs evolved by negative selection [67]. Ka/Ks analysis of the homologous *GmNCED* gene pairs revealed that all members of the *GmNCED* family engaged in purifying selection. This suggested that the *GmNCED* gene family was evolved to be highly conserved. Collinearity analysis revealed 6 collinear gene pairs in *P. apricot*, 6 in *P. salicina*, and 2 in *A. thaliana* [37]. Similarly, same numbers of *GmNCED* collinear gene pairs were identified, suggesting close genetic relationship among *GmNCED*s. In contrast, *GmNCED16* did not have any collinear gene pairs, suggesting that it might have a unique function or evolutionary history compared to the other *GmNCED* gene. But, no syntenic gene pairs were observed which implied a comparatively distant evolutionary relationship.

The uneven distribution of 16 *GmNCED* genes across 9 chromosomes implies potential functional diversification. Tandem, whole genome, and segmental duplications are the major driving mechanisms for the expansion of gene families in many plant species [68]. Eight gene pairs have undergone segmental duplication in *M. albus SRS* genes [69]. The prevalence of segmental duplications in this study among *GmNCED*s implies the significance in contributing to the expansion of the gene family. Furthermore, the *GmNCED* gene family has been conserved over time, likely due to its important functional role. In addition, the absence of tandem duplication events supported the hypothesis that segmental duplications were important in the expansion of this gene family. Subcellular localization of certain proteins is crucial to many plant biological processes and activities [70,71]. Most of NCED proteins are found in chloroplast in rice and other species [72–74]. For instance, in *Paeonia lactiflora* PlNCED1 and PlNCED2, found in the nucleus and cytoplasm respectively, functions in regulating transcription factors serving as an active enzyme for ABA biosynthesis [75]. Similarly, in this study, most of the GmNCED members were located in the cytoplasm and chloroplast suggesting their potential role in regulating ABA biosynthesis.

*Cis-*elements are crucial in the regulation of gene expression, particularly in response to drought stress and hormone signal transduction [76]. The presence of a wide variety of CAREs in the 5′ UTR region of *GmNCED*s implies their potential involvement in various stress responses and different plant hormone signaling pathways. The 5′ UTR region of *GmNCED6* and *GmNCED7* possessed CAREs including ARE, TCA-element, and ABRE, additionally, *GmNCED7* also possess CGTCA-motif as well as TGACG-motif relevant to MeJA-response. In addition, *MpNCED2* also included the CGTCA motif and the TGACG motif, which were important for the MeJA response [77]. Moreover, MBS CARE was found in *GmNCED* as drought inducibility response element [78]. The *GmNCED*s were mostly involved in the oxidation-reduction process, oxidoreductase activities, single organism metabolic process, dioxygenase activities as well as various catalytic activities in GO. Furthermore, GmNCED proteins play essential role in various catabolic, metabolic, and biosynthetic processes. Moreover, oxidoreductase activity was identified in *GmNCED*, involving with key role in photosynthesis, respiration, and detoxification as well as in plant defense systems [79].

TFs perform an important role in controlling a broad range of functions, including biotic and abiotic stress responses, promoting development and growth, metabolic regulation, and defense against microbial infections. ERF, MYB, bZIP, LBD, C2H2, GATA, and TALE are some of the major TFs in *GmNCED* [80–82]. ERF (Ethylene Response Factor) TF is responsible for both ethylene signaling and the response pathway in plants. It is characterized by a single AP2 domain [83]. Moreover, certain AP2/ERF TF families exhibit the engagement abscisic acid (ABA) and ethylene (ET) synthesis, which stimulate the expression of stress-responsive (SR) genes that are either dependent or independent on ABA and ET [84]. The expression of an ethylene response factor (SlERF5/ERF5) has a role in enhancing the resistance of tomato plants to salt and drought [85]. MYB TFs accounted for approximately 9% of total TFs in *Arabidopsis* [86]. Thus, MYB family is associated with defense and stress responses, circadian rhythm, cell identity and fate, seed and floral development, and primary and secondary metabolic control [86,87]. The basic leucine zipper (bZIP) TF family is involved in a wide range of plant biological processes, including embryonic development, seed maturation, organ differentiation, floral formation, both abiotic and biotic stressors, and vascular development [88,89]. The LBD play a vital role in various aspects of plant development, such as the growth, initiation, metabolic regulation, and secondary growth of leaves, stems, roots, and corollas. Furthermore, nitrogen metabolism and anthocyanin metabolism are both influenced by LBD genes [90–93]. ERF and GATA showcased strongest association with the *GmNCED* genes. While bZIP TF had the weakest association. In addition, predicted *GmNCED* genes and their associated TFs exhibited a diverse spectrum of expression patterns.

The 20-22 base pair long noncoding RNA regarded as miRNA is responsible for the regulation of gene expression by binding with target mRNA. The binding initiates the translational inhibition or breakdown of the former [94,95]. The regulation of gene expression mostly affects the growth and development of plants as well as cell division and differentiation in abiotic stress. The miRNAs are also involved in the hormonal signaling pathway [96–98]. The miRNA166 is responsible for plant growth and development including cell division, differentiation, and various organ development as well as regulating biotic and abiotic stress [99,100]. The miRNA166 in soybean revealed that it might regulate the hormone gibberellic acid metabolism (anabolism and catabolism) to control plant height [101]. On the other hand, miR482 was associated with drought and salt stress [102]. However, miR482 in the soybean was reported to take part in nodulation by nitrogen fixation bacteria as well as fungi to resist disease against pathogens [103]. In soybean, miR9752 was perhaps hyper-methylated in nodule formation condition [104].

The PPIs facilitate numerous biological mechanisms including cellular function coordination, signal transduction, and communication [105]. In this study, GmNCED protein interacted with CCD, D27, LCY1, Z-ISO, and BETA-OHASE2 protein family. CCD (Carotenoid Cleavage Dioxygenase), member of the CCO (carotenoid cleavage oxygenase) family, is closely related to NCED. They cleave carotenoids and form apocarotenoid molecules [106]. In response to stress signals, plants synthesize ABA, an apocarotenoid [107]. Iron-enriched D27 plant protein plays a crucial function in the synthesis of strigolactones hormone which is responsible for plant growth and development [108]. The carotenoid biosynthesis pathway relies on lycopene cyclases (LCY) enzymes, which are required to cyclize lycopene [109]. The RNA-seq has the central role in deciphering the complex analysis of gene expression in cells. It delivers an in-depth analysis of the genes that are actively being engaged and offers invaluable insight into the regulation and function of cells in different conditions such as various abiotic and biotic stress [110,111]. Most of the *GmNCED* showed down-regulation, however *GmNCED11* in particular showed up-regulation indicating potential role in dehydration stress. Under sodium salt stress treatment, certain genes in different time conditions showed higher expression and down-regulation as well. For example, *GmNCED2* showed up-regulation of expression in 6h and 12h whereas down-regulation at 3hr. *NCED* genes expression in cotton suggest the involvement of ABA biosynthesis [5]. Meanwhile, most of the *GmNCED* genes were down-regulated and few genes such as *GmNCED2*, *GmNCED14*, and *GmNCED15* showed up-regulation in drought stress. Moreover, soybean genes might play significant role in regulating drought signals [112]. Global warming and climate change posse severe threat to the production of food [113]. Hence, heat tolerant crop species are turning out to be obligatory to maintain their production at large in the adverse condition [114,115]. Soybean, economical crop species, contributes as dietary component (complete protein source), animal feed and oil [116]. Although soybean is a heat tolerant vegetative propagated oil crop, it is sensitive to high temperature during reproductive process such as seed growth, development and maturation. Thus, the consequences are shrinkage in seed quality, ineffective germination, higher chances of pathogen infection and ultimately damage to the economic value [117,118]. The RNA-seq analysis demonstrated the expression of *GmNCED* genes in the heat tolerant and conventional high yielding during 6hr imbibed seed, germinated seed and mature seed. The overexpression of *GmNCED15* during 6hr imbibed seed and mature seed showcased the heat stressed capability. Despite showing high expression in high yielding seed, *GmNCED2* didn’t exhibit up-regulation when introduced into the Huang heat tolerant seed. However, overexpression of *GmNCED13 and GmNCED14* during germinated seed stage provided the evidence that it might perform up regulation at heat stressed condition. ABA biosynthesis was involved in the seed development and germination stage during normal and heat stressed period [119]. The role of *NCED* gene in *Arabidopsis* revealed the seed specific expression in germination stage [13]. Hence, *GmNCED14* revealed substantial expression in managing the drought stress and seed germination in high temperature [120]. Over all, this study indicated the involvement of *GmNCED* in the regulation of various abiotic stress such as dehydration, salt, and drought. Moreover, *GmNCED* genes expression in seed germination at heat stressed condition involved ABA biosynthesis.

## 5. Conclusion

This study characterized 16 *GmNCED* genes containing the RPE65 domain allocated throughout the 9 chromosomes. Most of the *GmNCED* genes were located in the chloroplast and cytoplasm. Besides, *GmNCED*s were closely related to VvNCEDs according to the phylogenetic comparison. Diversification in gene structure and functional similarities within the *GmNCED* genes were discerned. *GmNCED* showcased segmental duplications and purifying selection process to establish their evolutionary significance. PPI exhibited the network within GmNCED. Most *GmNCED* genes were involved in biological functions such as metabolic, redox reaction, and biosynthetic in co-ordination with plant development and resistance to abiotic stress. CARE analysis revealed the involvement of *GmNCED* in drought stress. The miRNA regulated the expression of *GmNCED* against abiotic stress such as drought and slat as well as in seed germination stage. The RNA-seq data analysis confirmed the pivotal role of *GmNCED2*, *GmNCED11*, and *GmNCED12* under dehydration and sodium salt stress. Furthermore, *GmNCED14* and *GmNCED15* up-regulated their expression more frequently than other *GmNCED* genes under drought stress. Moreover, the higher expression of *GmNCED13* and *GmNCED14* in heat stressed germinated seed at high temperature regions indicated the induction of the ABA biosynthesis pathway. Hence, *GmNCED14* was up-regulated in both drought stress and seed germination stages elucidating the significance, it upholds in managing a challenged environment. Therefore, the findings from this study might provide a reliable and strong basis for functional characterization of *GmNCED* genes in wet lab conditions. Besides, this study contains useful information for the future breeding program aimed at improving the characteristics of this economically important soybean crop species.

## Supporting information

S1 Data Full-length protein sequences of *GmNCED* gene family. (TXT)

S2 Data Full-length protein sequences of *NCED* gene families of *G. max*, *R. chinensi, V. vinifer*, *A. thaliana*, *P. persica*, and *O. sativa* plant species for constructing a phylogenetic tree. (TXT)

S3 Data Full-length coding sequences of *GmNCED* gene families. (TXT)

S4 Data Full-length genomic sequences of *GmNCED* gene families. (TXT)

S5 Data The upstream promoter region (2.0 kb genomic sequences) of *GmNCED* gene families for the analysis of *cis*-acting regulatory elements. (TXT)

S6 Data Distribution of *GmNCED* gene family members among groups based on phylogenetic analysis. (DOCX)

S7 Data *In silico* predicted the number of introns and exons in *GmNCED* genes. (DOCX)

S8 Data Time of gene duplication estimated for different pairs of *GmNCED* genes based on Ka and Ks values. (XLSX)

S9 Data The predicted *cis*-acting regulatory elements of the upstream promoter region (2.0 kb genomic sequences) of the *GmNCED* gene. (XLSX)

S10 Data The GO analysis of *GmNCED* gene families for the identification of gene functions. (XLSX)

S11 Data The putative miRNA identification of *GmNCED* gene families. (XLSX)

S12 Data Protein-protein interactions of *GmNCED* protein families. (XLSX)

S13 Data Transcriptomic profiling of *GmNCED* genes in abiotic stresses, dehydration and salt. (XLSX)

S14 Data The transcriptomic profiling of GmNCED genes in drought stress in FPKM values. (XLSX)

S15 Data Transcriptomic profiling of *GmNCED* genes in seed developmental stage in heat stressed and control region. (XLSX)

S1 Fig Motif logos of *GmNCED* genes. (TIF)

S2 Fig Bubble plot of subcellular localization of *GmNCED* genes. (TIF)

## References

[1] Finkelstein R. Abscisic acid synthesis and response. The Arabidopsis book/American society of plant biologists. 2013;11. 10.1199/tab.0166 PMC3833200 24273463

[2] Nambara E, Marion-Poll A. Abscisic acid biosynthesis and catabolism. Annu Rev Plant Biol. 2005;56:165–85. doi: 10.1146/annurev.arplant.56.032604.144046 15862093

[3] Schwartz SH, Tan BC, Gage DA, Zeevaart JA, McCarty DR. Specific oxidative cleavage of carotenoids by VP14 of maize. Science. 1997;276(5320):1872–4. doi: 10.1126/science.276.5320.1872 9188535

[4] Taylor IB, Burbidge A, Thompson AJ. Control of abscisic acid synthesis. J Exp Bot. 2000;51(350):1563–74. doi: 10.1093/jexbot/51.350.1563 11006307

[5] Li Q, Yu X, Chen L, Zhao G, Li S, Zhou H, et al. Genome-wide identification and expression analysis of the NCED family in cotton (Gossypium hirsutum L.). PLoS One. 2021;16(2):e0246021. doi: 10.1371/journal.pone.0246021 33630882 PMC7906304

[6] Yue X-Q, Zhang Y, Yang C-K, Li J-G, Rui X, Ding F, et al. Genome-wide identification and expression analysis of carotenoid cleavage oxygenase genes in Litchi (Litchi chinensis Sonn.). BMC Plant Biol. 2022;22(1):394. doi: 10.1186/s12870-022-03772-w 35945492 PMC9361530

[7] Zhao X-L, Yang Y-L, Xia H-X, Li Y. Genome-wide analysis of the carotenoid cleavage dioxygenases gene family in Forsythia suspensa: Expression profile and cold and drought stress responses. Front Plant Sci. 2022;13:998911. doi: 10.3389/fpls.2022.998911 36204048 PMC9531035

[8] Tan BC, Schwartz SH, Zeevaart JA, McCarty DR. Genetic control of abscisic acid biosynthesis in maize. Proc Natl Acad Sci U S A. 1997;94(22):12235–40. doi: 10.1073/pnas.94.22.12235 9342392 PMC23760

[9] Tan B-C, Joseph LM, Deng W-T, Liu L, Li Q-B, Cline K, et al. Molecular characterization of the Arabidopsis 9-cis epoxycarotenoid dioxygenase gene family. Plant J. 2003;35(1):44–56. doi: 10.1046/j.1365-313x.2003.01786.x 12834401

[10] Wang X, Liu F, Shi X, Wang X, Ji X, Wang Z. Evolution and expression of NCED family genes in Vitis vinifera. Chinese Bulletin of Botany. 2019;54(4):474.

[11] Chernys JT, Zeevaart JA. Characterization of the 9-cis-epoxycarotenoid dioxygenase gene family and the regulation of abscisic acid biosynthesis in avocado. Plant Physiol. 2000;124(1):343–53. doi: 10.1104/pp.124.1.343 10982448 PMC59148

[12] Zhu G, Ye N, Zhang J. Glucose-induced delay of seed germination in rice is mediated by the suppression of ABA catabolism rather than an enhancement of ABA BIOSYNTHESIS. Plant and Cell Physiology. 2009;50(3):644–51. doi: 10.1093/pcp/pcp022 19208695

[13] Lefebvre V, North H, Frey A, Sotta B, Seo M, Okamoto M, et al. Functional analysis of Arabidopsis NCED6 and NCED9 genes indicates that ABA synthesized in the endosperm is involved in the induction of seed dormancy. Plant J. 2006;45(3):309–19. doi: 10.1111/j.1365-313X.2005.02622.x 16412079

[14] Martínez-Andújar C, Ordiz MI, Huang Z, Nonogaki M, Beachy RN, Nonogaki H. Induction of 9-cis-epoxycarotenoid dioxygenase in Arabidopsis thaliana seeds enhances seed dormancy. Proc Natl Acad Sci U S A. 2011;108(41):17225–9. doi: 10.1073/pnas.1112151108 21969557 PMC3193201

[15] Frey A, Effroy D, Lefebvre V, Seo M, Perreau F, Berger A, et al. Epoxycarotenoid cleavage by NCED5 fine-tunes ABA accumulation and affects seed dormancy and drought tolerance with other NCED family members. Plant J. 2012;70(3):501–12. doi: 10.1111/j.1365-313X.2011.04887.x 22171989

[16] Matilla AJ, Carrillo-Barral N, Rodríguez-Gacio M del C. An update on the role of NCED and CYP707A ABA metabolism genes in seed dormancy induction and the response to after-ripening and nitrate. J Plant Growth Regul. 2014;34(2):274–93. doi: 10.1007/s00344-014-9464-7

[17] Endo A, Koshiba T, Kamiya Y, Nambara E. Vascular system is a node of systemic stress responses: Competence of the cell to synthesize abscisic acid and its responsiveness to external cues. Plant Signal Behav. 2008;3(12):1138–40. doi: 10.4161/psb.3.12.7145 19704460 PMC2634481

[18] Hao G-P, Zhang X-H, Wang Y-Q, Wu Z-Y, Huang C-L. Nucleotide variation in the NCED3 region of Arabidopsis thaliana and its association study with abscisic acid content under drought stress. J Integr Plant Biol. 2009;51(2):175–83. doi: 10.1111/j.1744-7909.2008.00786.x 19200156

[19] Ye N, Jia L, Zhang J. ABA signal in rice under stress conditions. Rice (N Y). 2012;5(1):1. doi: 10.1186/1939-8433-5-1 24764501 PMC3834477

[20] Hwang S-G, Lee C-Y, Tseng C-S. Heterologous expression of rice 9-cis-epoxycarotenoid dioxygenase 4 (OsNCED4) in Arabidopsis confers sugar oversensitivity and drought tolerance. Bot Stud. 2018;59(1):2. doi: 10.1186/s40529-018-0219-9 29335785 PMC5768580

[21] Hwang S-G, Chen H-C, Huang W-Y, Chu Y-C, Shii C-T, Cheng W-H. Ectopic expression of rice OsNCED3 in Arabidopsis increases ABA level and alters leaf morphology. Plant Science. 2010;178(1):12–22. doi: 10.1016/j.plantsci.2009.09.014

[22] Huang H, Ullah F, Zhou D-X, Yi M, Zhao Y. Mechanisms of ROS regulation of plant development and stress responses. Front Plant Sci. 2019;10:800. doi: 10.3389/fpls.2019.00800 31293607 PMC6603150

[23] Krasensky J, Jonak C. Drought, salt, and temperature stress-induced metabolic rearrangements and regulatory networks. J Exp Bot. 2012;63(4):1593–608. doi: 10.1093/jxb/err460 22291134 PMC4359903

[24] Hamel L-P, Nicole M-C, Duplessis S, Ellis BE. Mitogen-activated protein kinase signaling in plant-interacting fungi: distinct messages from conserved messengers. Plant Cell. 2012;24(4):1327–51. doi: 10.1105/tpc.112.096156 22517321 PMC3398478

[25] Kim T-H, Böhmer M, Hu H, Nishimura N, Schroeder JI. Guard cell signal transduction network: advances in understanding abscisic acid, CO2, and Ca2+ signaling. Annu Rev Plant Biol. 2010;61:561–91. doi: 10.1146/annurev-arplant-042809-112226 20192751 PMC3056615

[26] Muhammad Aslam M, Waseem M, Jakada BH, Okal EJ, Lei Z, Saqib HSA, et al. Mechanisms of Abscisic Acid-Mediated Drought Stress Responses in Plants. Int J Mol Sci. 2022;23(3):1084. doi: 10.3390/ijms23031084 35163008 PMC8835272

[27] Verma V, Ravindran P, Kumar P. Plant hormone-mediated regulation of stress responses. Journal of Plant Biology. 2016;16:1–10. 10.1186/s12870-016-0771-y PMC4831116 27079791

[28] Deak KI, Malamy J. Osmotic regulation of root system architecture. Plant J. 2005;43(1):17–28. doi: 10.1111/j.1365-313X.2005.02425.x 15960613

[29] Anai T. Potential of a mutant-based reverse genetic approach for functional genomics and molecular breeding in soybean. Breed Sci. 2012;61(5):462–7. doi: 10.1270/jsbbs.61.462 23136486 PMC3406801

[30] Tran L-SP, Mochida K. Identification and prediction of abiotic stress responsive transcription factors involved in abiotic stress signaling in soybean. Plant Signal Behav. 2010;5(3):255–7. doi: 10.4161/psb.5.3.10550 20023425 PMC2881270

[31] Zhang Y, Xu J, Li R, Ge Y, Li Y, Li R. Plants’ response to abiotic stress: mechanisms and strategies. Int J Mol Sci. 2023;24(13):10915. doi: 10.3390/ijms241310915 37446089 PMC10341657

[32] Goodstein DM, Shu S, Howson R, Neupane R, Hayes RD, Fazo J, et al. Phytozome: a comparative platform for green plant genomics. Nucleic Acids Res. 2012;40(Database issue):D1178-86. doi: 10.1093/nar/gkr944 22110026 PMC3245001

[33] Letunic I, Khedkar S, Bork P. SMART: recent updates, new developments and status in 2020. Nucleic Acids Res. 2021;49(D1):D458–60. doi: 10.1093/nar/gkaa937 33104802 PMC7778883

[34] Lu S, Wang J, Chitsaz F, Derbyshire MK, Geer RC, Gonzales NR, et al. CDD/SPARCLE: the conserved domain database in 2020. Nucleic Acids Res. 2020;48(D1):D265–8. doi: 10.1093/nar/gkz991 31777944 PMC6943070

[35] Garg VK, Avashthi H, Tiwari A, Jain PA, Ramkete PW, Kayastha AM, et al. MFPPI - Multi FASTA ProtParam Interface. Bioinformation. 2016;12(2):74–7. doi: 10.6026/97320630012074 28104964 PMC5237651

[36] Chen L, Dong X, Yang H, Chai Y, Xia Y, Tian L, et al. Cytosolic disproportionating enzyme2 is essential for pollen germination and pollen tube elongation in rice. Plant Physiol. 2023;191(1):96–109. doi: 10.1093/plphys/kiac496 36282529 PMC9806659

[37] Chen K, Li X, Guo X, Yang L, Qiu L, Liu W, et al. Genome-wide identification and expression profiling of the nced gene family in cold stress response of prunus mume Siebold & Zucc. Horticulturae. 2023;9(7):839. doi: 10.3390/horticulturae9070839

[38] Kumar S, Stecher G, Li M, Knyaz C, Tamura K. MEGA X: molecular evolutionary genetics analysis across computing platforms. Mol Biol Evol. 2018;35(6):1547–9. doi: 10.1093/molbev/msy096 29722887 PMC5967553

[39] Thompson JD, Gibson TJ, Higgins DG. Multiple sequence alignment using ClustalW and ClustalX. Curr Protoc Bioinformatics. 2002;Chapter 2:Unit 2.3. doi: 10.1002/0471250953.bi0203s00 18792934

[40] Letunic I, Bork P. Interactive Tree Of Life (iTOL) v5: an online tool for phylogenetic tree display and annotation. Nucleic Acids Res. 2021;49(W1):W293–6. doi: 10.1093/nar/gkab301 33885785 PMC8265157

[41] Guo A-Y, Zhu Q-H, Chen X, Luo J-C. GSDS: a gene structure display server. Yi Chuan. 2007;29(8):1023–6. doi: 10.1360/yc-007-1023 17681935

[42] Chen C, Chen H, Zhang Y, Thomas HR, Frank MH, He Y, et al. TBtools: an integrative toolkit developed for interactive analyses of big biological data. Mol Plant. 2020;13(8):1194–202. doi: 10.1016/j.molp.2020.06.009 32585190

[43] Bailey TL, Johnson J, Grant CE, Noble WS. The MEME suite. Nucleic Acids Res. 2015;43(W1):W39-49. doi: 10.1093/nar/gkv416 25953851 PMC4489269

[44] Wang D, Zhang Y, Zhang Z, Zhu J, Yu J. KaKs_Calculator 2.0: a toolkit incorporating gamma-series methods and sliding window strategies. Genomics Proteomics Bioinformatics. 2010;8(1):77–80. doi: 10.1016/S1672-0229(10)60008-3 20451164 PMC5054116

[45] Lynch M, Conery JS. The evolutionary fate and consequences of duplicate genes. Science. 2000;290(5494):1151–5. doi: 10.1126/science.290.5494.1151 11073452

[46] Chao J, Li Z, Sun Y, Aluko OO, Wu X, Wang Q, et al. MG2C: a user-friendly online tool for drawing genetic maps. Mol Hortic. 2021;1(1):16. doi: 10.1186/s43897-021-00020-x 37789491 PMC10514940

[47] Horton P, Park K-J, Obayashi T, Fujita N, Harada H, Adams-Collier CJ, et al. WoLF PSORT: protein localization predictor. Nucleic Acids Res. 2007;35(Web Server issue):W585-7. doi: 10.1093/nar/gkm259 17517783 PMC1933216

[48] Team RCJC. RA language and environment for statistical computing, R Foundation for Statistical. 2020.

[49] Rombauts S, Déhais P, Van Montagu M, Rouzé P. PlantCARE, a plant cis-acting regulatory element database. Nucleic Acids Res. 1999;27(1):295–6. doi: 10.1093/nar/27.1.295 9847207 PMC148162

[50] Tian F, Yang D-C, Meng Y-Q, Jin J, Gao G. PlantRegMap: charting functional regulatory maps in plants. Nucleic Acids Res. 2020;48(D1):D1104–13. doi: 10.1093/nar/gkz1020 31701126 PMC7145545

[51] Xie J, Chen Y, Cai G, Cai R, Hu Z, Wang H. Tree Visualization By One Table (tvBOT): a web application for visualizing, modifying and annotating phylogenetic trees. Nucleic Acids Res. 2023;51(W1):W587–92. doi: 10.1093/nar/gkad359 37144476 PMC10320113

[52] Shannon P, Markiel A, Ozier O, Baliga NS, Wang JT, Ramage D, et al. Cytoscape: a software environment for integrated models of biomolecular interaction networks. Genome Res. 2003;13(11):2498–504. doi: 10.1101/gr.1239303 14597658 PMC403769

[53] Kozomara A, Birgaoanu M, Griffiths-Jones S. miRBase: from microRNA sequences to function. NAR. 2019;47(D1):D155–62. 30423142 10.1093/nar/gky1141 PMC6323917

[54] Dai X, Zhao PX. psRNATarget: a plant small RNA target analysis server. Nucleic Acids Res. 2011;39(Web Server issue):W155-9. doi: 10.1093/nar/gkr319 21622958 PMC3125753

[55] Szklarczyk D, Gable AL, Lyon D, Junge A, Wyder S, Huerta-Cepas J, et al. STRING v11: protein-protein association networks with increased coverage, supporting functional discovery in genome-wide experimental datasets. Nucleic Acids Res. 2019;47(D1):D607–13. doi: 10.1093/nar/gky1131 30476243 PMC6323986

[56] Yang X, Hu Q, Zhao Y, Chen Y, Li C, He J, et al. Identification of GmPT proteins and investigation of their expressions in response to abiotic stress in soybean. Planta. 2024;259(4):76. doi: 10.1007/s00425-024-04348-8 38418674

[57] Gillman JD, Biever JJ, Ye S, Spollen WG, Givan SA, Lyu Z, et al. A seed germination transcriptomic study contrasting two soybean genotypes that differ in terms of their tolerance to the deleterious impacts of elevated temperatures during seed fill. BMC Res Notes. 2019;12(1):522. doi: 10.1186/s13104-019-4559-7 31426836 PMC6700996

[58] Bolger AM, Lohse M, Usadel B. Trimmomatic: a flexible trimmer for Illumina sequence data. Bioinformatics. 2014;30(15):2114–20. doi: 10.1093/bioinformatics/btu170 24695404 PMC4103590

[59] Dobin A, Gingeras TR. Mapping RNA-seq Reads with STAR. Curr Protoc Bioinformatics. 2015;51:11.14.1-11.14.19. doi: 10.1002/0471250953.bi1114s51 26334920 PMC4631051

[60] Li H, Handsaker B, Wysoker A, Fennell T, Ruan J, Homer N, et al. The sequence alignment/map format and SAMtools. Bioinformatics. 2009;25(16):2078–9. doi: 10.1093/bioinformatics/btp352 19505943 PMC2723002

[61] Li B, Dewey CN. RSEM: accurate transcript quantification from RNA-Seq data with or without a reference genome. BMC Bioinformatics. 2011;12:323. doi: 10.1186/1471-2105-12-323 21816040 PMC3163565

[62] Shen X-X, Salichos L, Rokas A. A Genome-scale investigation of how sequence, function, and tree-based gene properties influence phylogenetic inference. Genome Biol Evol. 2016;8(8):2565–80. doi: 10.1093/gbe/evw179 27492233 PMC5010910

[63] Koonin EV, Csuros M, Rogozin IB. Whence genes in pieces: reconstruction of the exon-intron gene structures of the last eukaryotic common ancestor and other ancestral eukaryotes. Wiley Interdiscip Rev RNA. 2013;4(1):93–105. doi: 10.1002/wrna.1143 23139082

[64] Colquhoun IJ, Le Gall G, Elliott KA, Mellon FA, Michael AJ. Shall I compare thee to a GM potato?. Trends Genet. 2006;22(10):525–8. doi: 10.1016/j.tig.2006.08.002 16904227

[65] Heidari P, Puresmaeli F, Mora-Poblete F. Genome-wide identification and molecular evolution of the magnesium transporter (MGT) gene family in citrullus lanatus and cucumis sativus. Agronomy. 2022;12(10):2253. doi: 10.3390/agronomy12102253

[66] Alternative EoE-ISa, Splicing.

[67] Liu J, Yuan X, Quan S, Zhang M, Kang C, Guo C, et al. Genome-wide identification and expression analysis of NCED gene family in pear and its response to exogenous gibberellin and paclobutrazol. Int J Mol Sci. 2023;24(8):7566. doi: 10.3390/ijms24087566 37108747 PMC10144387

[68] Huang H, Song J, Feng Y, Zheng L, Chen Y, Luo K. Genome-wide identification and expression analysis of the SHI-related sequence family in cassava. Genes (Basel). 2023;14(4):870. doi: 10.3390/genes14040870 37107628 PMC10138042

[69] Ma B, Nian L, Ain NU, Liu X, Yang Y, Zhu X, et al. Genome-wide identification and expression profiling of the SRS gene family in Melilotus albus reveals functions in various stress conditions. Plants (Basel). 2022;11(22):3101. doi: 10.3390/plants11223101 36432830 PMC9693462

[70] Ehrlich JS, Hansen MDH, Nelson WJ. Spatio-temporal regulation of Rac1 localization and lamellipodia dynamics during epithelial cell-cell adhesion. Dev Cell. 2002;3(2):259–70. doi: 10.1016/s1534-5807(02)00216-2 12194856 PMC3369831

[71] Glory E, Murphy RF. Automated subcellular location determination and high-throughput microscopy. Dev Cell. 2007;12(1):7–16. doi: 10.1016/j.devcel.2006.12.007 17199037

[72] Lee Y-I, Chen M-C, Lin L, Chung M-C, Leu W-M. Increased expression of 9-cis-epoxycarotenoid dioxygenase, PtNCED1, associated with inhibited seed germination in a terrestrial orchid, Phaius tankervilliae. Front Plant Sci. 2018;9:1043. doi: 10.3389/fpls.2018.01043 30065747 PMC6056907

[73] Chen Y, Xiang Z, Liu M, Wang S, Zhang L, Cai D, et al. ABA biosynthesis gene OsNCED3 contributes to preharvest sprouting resistance and grain development in rice. Plant Cell Environ. 2023;46(4):1384–401. doi: 10.1111/pce.14480 36319615

[74] Jia Y, Liu J, Bai Z, Ding K, Li H, Liang Z. Cloning and functional characterization of the SmNCED3 in Salvia miltiorrhiza. Acta Physiol Plant. 2018;40(7):. doi: 10.1007/s11738-018-2704-x

[75] Fei R, Guan S, Duan S, Ge J, Sun T, Sun X. Elucidating biological functions of 9-cis-epoxycarotenoid dioxygenase genes involved in seed dormancy in Paeonia lactiflora. Plants (Basel). 2023;12(4):710. doi: 10.3390/plants12040710 36840058 PMC9967950

[76] Wittkopp PJ, Kalay G. Cis-regulatory elements: molecular mechanisms and evolutionary processes underlying divergence. Nat Rev Genet. 2011;13(1):59–69. doi: 10.1038/nrg3095 22143240

[77] Xia H, Wu S, Ma F. Cloning and expression of two 9-cis-epoxycarotenoid dioxygenase genes during fruit development and under stress conditions from Malus. Mol Biol Rep. 2014;41(10):6795–802. doi: 10.1007/s11033-014-3565-z 25043349

[78] Yang X, Guo T, Li J, Chen Z, Guo B, An X. Genome-wide analysis of the MYB-related transcription factor family and associated responses to abiotic stressors in Populus. Int J Biol Macromol. 2021;191:359–76. doi: 10.1016/j.ijbiomac.2021.09.042 34534587

[79] Habte M, Beyene E. Biological application and disease of oxidoreductase enzymes. Oxidoreductase: IntechOpen; 2020.

[80] Lutova LA, Dodueva IE, Lebedeva MA, Tvorogova VE. Transcription factors in developmental genetics and the evolution of higher plants. Genetika. 2015;51(5):539–57. doi: 10.1134/s1022795415030084 26137635

[81] Mengiste T, Chen X, Salmeron J, Dietrich R. The BOTRYTIS SUSCEPTIBLE1 gene encodes an R2R3MYB transcription factor protein that is required for biotic and abiotic stress responses in Arabidopsis. Plant Cell. 2003;15(11):2551–65. doi: 10.1105/tpc.014167 14555693 PMC280560

[82] Meshi T, Iwabuchi M. Plant transcription factors. Plant Cell Physiol. 1995;36(8):1405–20. 8589926

[83] Müller M, Munné-Bosch S. Ethylene response factors: a key regulatory hub in hormone and stress signaling. Plant Physiol. 2015;169(1):32–41. doi: 10.1104/pp.15.00677 26103991 PMC4577411

[84] Xie Z, Nolan T, Jiang H, Yin Y. AP2/ERF transcription factor regulatory networks in hormone and abiotic stress responses in Arabidopsis. Frontiers in Plant Science. 2019;10:228. 30873200 10.3389/fpls.2019.00228 PMC6403161

[85] Pan Y, Seymour GB, Lu C, Hu Z, Chen X, Chen G. An ethylene response factor (ERF5) promoting adaptation to drought and salt tolerance in tomato. Plant Cell Rep. 2012;31(2):349–60. doi: 10.1007/s00299-011-1170-3 22038370

[86] Cao Y, Li K, Li Y, Zhao X, Wang L. MYB transcription factors as regulators of secondary metabolism in plants. Biology (Basel). 2020;9(3):61. doi: 10.3390/biology9030061 32213912 PMC7150910

[87] Ramya M, Kwon OK, An HR, Park PM, Baek YS, Park PH. Floral scent: regulation and role of MYB transcription factors. Phytochemistry Letters. 2017;19:114–20. doi: 10.1016/j.phytol.2016.12.015

[88] Wei K, Chen J, Wang Y, Chen Y, Chen S, Lin Y, et al. Genome-wide analysis of bZIP-encoding genes in maize. DNA Res. 2012;19(6):463–76. doi: 10.1093/dnares/dss026 23103471 PMC3514857

[89] Jakoby M, Weisshaar B, Dröge-Laser W, Vicente-Carbajosa J, Tiedemann J, Kroj T, et al. bZIP transcription factors in Arabidopsis. Trends Plant Sci. 2002;7(3):106–11. doi: 10.1016/s1360-1385(01)02223-3 11906833

[90] Fan M, Xu C, Xu K, Hu Y. LATERAL ORGAN BOUNDARIES DOMAIN transcription factors direct callus formation in Arabidopsis regeneration. Cell Res. 2012;22(7):1169–80. doi: 10.1038/cr.2012.63 22508267 PMC3391013

[91] Rubin G, Tohge T, Matsuda F, Saito K, Scheible W-R. Members of the LBD family of transcription factors repress anthocyanin synthesis and affect additional nitrogen responses in Arabidopsis. Plant Cell. 2009;21(11):3567–84. doi: 10.1105/tpc.109.067041 19933203 PMC2798321

[92] Shuai B, Reynaga-Peña CG, Springer PS. The lateral organ boundaries gene defines a novel, plant-specific gene family. Plant Physiol. 2002;129(2):747–61. doi: 10.1104/pp.010926 12068116 PMC161698

[93] Majer C, Hochholdinger F. Defining the boundaries: structure and function of LOB domain proteins. Trends Plant Sci. 2011;16(1):47–52. doi: 10.1016/j.tplants.2010.09.009 20961800

[94] Bajczyk M, Jarmolowski A, Jozwiak M, Pacak A, Pietrykowska H, Sierocka I, et al. Recent insights into plant miRNA biogenesis: multiple layers of miRNA level regulation. Plants (Basel). 2023;12(2):342. doi: 10.3390/plants12020342 36679055 PMC9864873

[95] Samad AFA, Sajad M, Nazaruddin N, Fauzi IA, Murad AMA, Zainal Z, et al. MicroRNA and transcription factor: key players in plant regulatory network. Front Plant Sci. 2017;8 565. doi: 10.3389/fpls.2017.00565 28446918 PMC5388764

[96] Dong Q, Hu B, Zhang C. microRNAs and their roles in plant development. Front Plant Sci. 2022;13:824240. doi: 10.3389/fpls.2022.824240 35251094 PMC8895298

[97] Yang Y, Huang J, Sun Q, Wang J, Huang L, Fu S, et al. microRNAs: key players in plant response to metal toxicity. Int J Mol Sci. 2022;23(15):8642. doi: 10.3390/ijms23158642 35955772 PMC9369385

[98] Ahmad HM, Wang X, Ijaz M, , Oranab S, Ali MA, et al. Molecular aspects of MicroRNAs and phytohormonal signaling in response to drought stress: a review. Curr Issues Mol Biol. 2022;44(8):3695–710. doi: 10.3390/cimb44080253 36005149 PMC9406886

[99] Yadav A, Kumar S, Verma R, Lata C, Sanyal I, Rai S. microRNA 166: an evolutionarily conserved stress biomarker in land plants targeting HD-ZIP family. Physiol Mol Biol Plants. 2021;27(11):2471–85. doi: 10.1007/s12298-021-01096-x 34924705 PMC8639965

[100] Asha S, Nisha J, Soniya EV. In silico characterisation and phylogenetic analysis of two evolutionarily conserved miRNAs (miR166 and miR171) from black pepper (Piper nigrum L.). Plant Mol Biol Rep. 2012;31(3):707–18. doi: 10.1007/s11105-012-0532-5

[101] Zhao C, Ma J, Zhang Y, Yang S, Feng X, Yan J. The miR166 mediated regulatory module controls plant height by regulating gibberellic acid biosynthesis and catabolism in soybean. J Integr Plant Biol. 2022;64(5):995–1006. doi: 10.1111/jipb.13253 35312167

[102] An Y, Su H, Niu Q, Yin S. Integrated analysis of coding and non-coding RNAs reveals the molecular mechanism underlying salt stress response in Medicago truncatula. Front Plant Sci. 2022;13 891361. doi: 10.3389/fpls.2022.891361 35519807 PMC9064118

[103] Eren AH, İlhan E, İnal B. MiR482 and its isoforms in plants. 2016.

[104] Piya S, Lopes-Caitar V, Kim W, Pantalone V, Krishnan H, Hewezi T. Hypermethylation of miRNA genes during nodule development. Journal of Functional Integrative Biology. 2021;8:616–23. 10.3389/fmolb.2021.616623 PMC8076613 33928115

[105] Westermarck J, Ivaska J, Corthals GL. Identification of protein interactions involved in cellular signaling. Mol Cell Proteomics. 2013;12(7):1752–63. doi: 10.1074/mcp.R113.027771 23481661 PMC3708163

[106] Priya R, Sneha P, Dass JFP, Doss C GP, Manickavasagam M, Siva R. Exploring the codon patterns between CCD and NCED genes among different plant species. Comput Biol Med. 2019;114:103449. doi: 10.1016/j.compbiomed.2019.103449 31568976

[107] Hou X, Rivers J, León P, McQuinn RP, Pogson BJ. Synthesis and function of apocarotenoid signals in plants. Trends Plant Sci. 2016;21(9):792–803. doi: 10.1016/j.tplants.2016.06.001 27344539

[108] Tolnai Z, Sharma H, Soós V. D27-like carotenoid isomerases: at the crossroads of strigolactone and abscisic acid biosynthesis. J Exp Bot. 2024;75(4):1148–58. doi: 10.1093/jxb/erad475 38006582

[109] Koc I, Filiz E, Tombuloglu H. Comparative analysis of plant lycopene cyclases. Comput Biol Chem. 2015;58:81–92. doi: 10.1016/j.compbiolchem.2015.06.001 26092704

[110] Wang Z, Gerstein M, Snyder M. RNA-Seq: a revolutionary tool for transcriptomics. Nat Rev Genet. 2009;10(1):57–63. doi: 10.1038/nrg2484 19015660 PMC2949280

[111] Yu G, Zhou Y, Yu J, Hu X, Tang Y, Yan H, et al. Author correction: transcriptome and digital gene expression analysis unravels the novel mechanism of early flowering in Angelica sinensis. Sci Rep. 2020;10(1):1888. doi: 10.1038/s41598-020-58024-4 32005842 PMC6994468

[112] Marcolino-Gomes J, Rodrigues FA, Fuganti-Pagliarini R, Nakayama TJ, Ribeiro Reis R, Bouças Farias JR, et al. Transcriptome-wide identification of reference genes for expression analysis of soybean responses to drought stress along the day. PLoS One. 2015;10(9):e0139051. doi: 10.1371/journal.pone.0139051 26407065 PMC4583485

[113] Wheeler T, von Braun J. Climate change impacts on global food security. Science. 2013;341(6145):508–13. doi: 10.1126/science.1239402 23908229

[114] Bita CE, Gerats T. Plant tolerance to high temperature in a changing environment: scientific fundamentals and production of heat stress-tolerant crops. Front Plant Sci. 2013;4 273. doi: 10.3389/fpls.2013.00273 23914193 PMC3728475

[115] Hasanuzzaman M, Nahar K, Alam MM, Roychowdhury R, Fujita M. Physiological, biochemical, and molecular mechanisms of heat stress tolerance in plants. Int J Mol Sci. 2013;14(5):9643–84. doi: 10.3390/ijms14059643 23644891 PMC3676804

[116] Liu K. Soybean: Overview. Reference Module in Food Science. Elsevier; 2016.

[117] Chebrolu K, Fritschi F, Ye S, Krishnan H, Smith J, Gillman J. Impact of heat stress during seed development on soybean seed metabolome. Journal of Plant Physiology. 2016;12:1–14.

[118] Jianing G, Yuhong G, Yijun G, Rasheed A, Qian Z, Zhiming X, et al. Improvement of heat stress tolerance in soybean (Glycine max L), by using conventional and molecular tools. Front Plant Sci. 2022;13:993189. doi: 10.3389/fpls.2022.993189 36226280 PMC9549248

[119] Sano N, Marion-Poll A. ABA metabolism and homeostasis in seed dormancy and germination. Int J Mol Sci. 2021;22(10):5069. doi: 10.3390/ijms22105069 34064729 PMC8151144

[120] Huang Y, Jiao Y, Yang S, Mao D, Wang F, Chen L, et al. SiNCED1, a 9-cis-epoxycarotenoid dioxygenase gene in Setaria italica, is involved in drought tolerance and seed germination in transgenic Arabidopsis. Front Plant Sci. 2023;14:1121809. doi: 10.3389/fpls.2023.1121809 36968367 PMC10034083

