# Supplementary material for: Genome-wide identification and characterization of NCED gene family in soybean (Glycine max L.) and their expression profiles in response to various abiotic stress treatments
Source: PLoS One. 2025 Mar 25;20(3):e0319952. doi: 10.1371/journal.pone.0319952 (PMC11936224; doi:10.1371/journal.pone.0319952)
Supplement: S6 Data — (DOCX) [file pone.0319952.s006.docx]

**S6 Data.** Distribution of *GmNCED* gene family members among groups based on phylogenetic analysis.

| **Group name** | **Number of genes** | **Gene ID** |
| --- | --- | --- |
| A | 2 | *GmNCED5, GmNCED9* |
| B | 2 | *GmNCED1, GmNCED12* |
| C | 4 | *GmNCED3, GmNCED4, GmNCED7, GmNCED8* |
| D | 1 | *GmNCED2* |
| E | 3 | *GmNCED13, GmNCED14, GmNCED15* |
| F | 4 | *GmNCED6, GmNCED10, GmNCED11, GmNCED16* |
