# Supplementary material for: Genome-wide identification and characterization of NCED gene family in soybean (Glycine max L.) and their expression profiles in response to various abiotic stress treatments
Source: PLoS One. 2025 Mar 25;20(3):e0319952. doi: 10.1371/journal.pone.0319952 (PMC11936224; doi:10.1371/journal.pone.0319952)
Supplement: S7 Data — (DOCX) [file pone.0319952.s007.docx]

**S7 Data.** *In silico* predicted the number of introns and exons in *GmNCED* genes.

| **Group name** | **Gene name** | **Source accession** | **Intron** | **Exons** |
| --- | --- | --- | --- | --- |
| A | *GmNCED5* | Glyma.04G084100 | 5 | 6 |
|  | *GmNCED9* | Glyma.06G085800 | 5 | 6 |
| B | *GmNCED1* | Glyma.01G073200 | 6 | 7 |
|  | *GmNCED12* | Glyma.11G161947 | 6 | 7 |
| C | *GmNCED3* | Glyma.04G083500 | 12 | 13 |
|  | *GmNCED4* | Glyma.04G083600 | 7 | 8 |
|  | *GmNCED7* | Glyma.06G085000 | 12 | 13 |
|  | *GmNCED8* | Glyma.06G085100 | 12 | 13 |
| D | *GmNCED2* | Glyma.01G154900 | 1 | 2 |
| E | *GmNCED13* | Glyma.12G236650 | 13 | 14 |
|  | *GmNCED14* | Glyma.12G236700 | 7 | 8 |
|  | *GmNCED15* | Glyma.13G202200 | 6 | 7 |
| F | *GmNCED6* | Glyma.05G140900 | 0 | 1 |
|  | *GmNCED10* | Glyma.08G096200 | 0 | 1 |
|  | *GmNCED11* | Glyma.08G176300 | 0 | 1 |
|  | *GmNCED16* | Glyma.15G250100 | 0 | 1 |
