# Supplementary figures and images for: Genome-wide identification and characterization of NCED gene family in soybean (Glycine max L.) and their expression profiles in response to various abiotic stress treatments
Source: PLoS One. 2025 Mar 25;20(3):e0319952. doi: 10.1371/journal.pone.0319952 (PMC11936224; doi:10.1371/journal.pone.0319952)

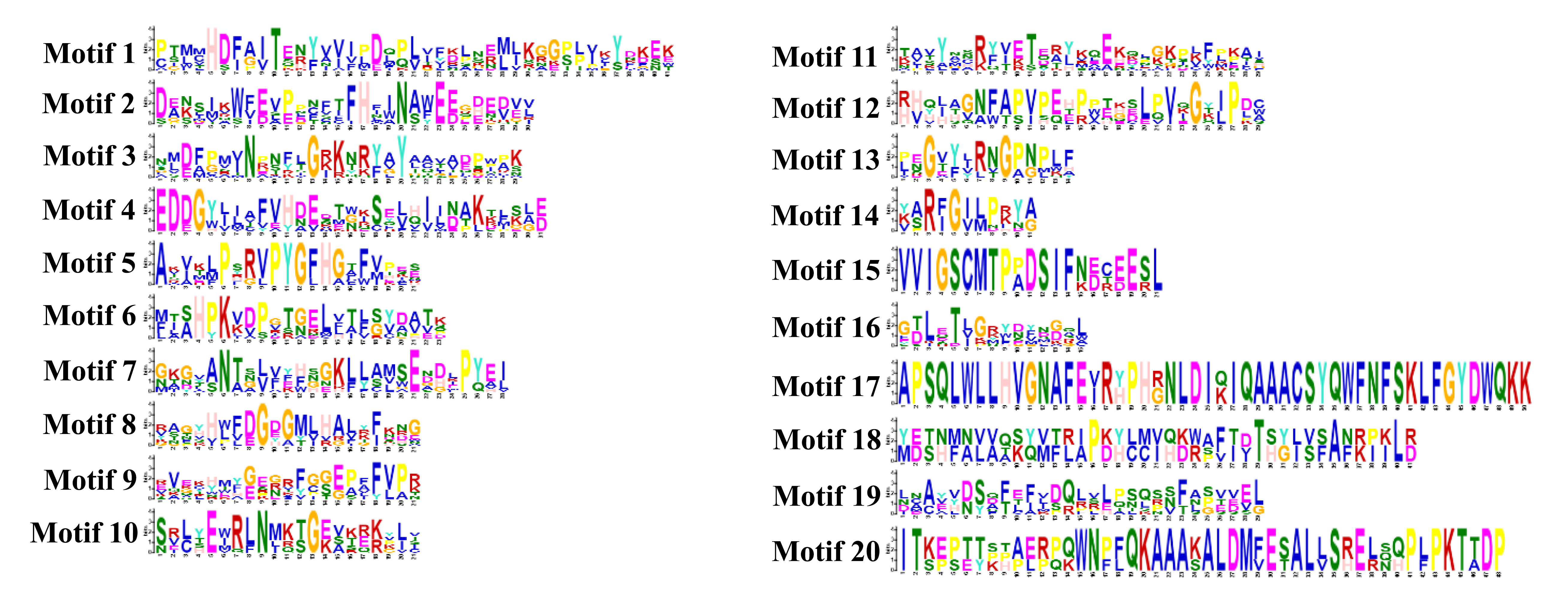

Supplement: S1 Fig — (TIF) [file pone.0319952.s016.tif]

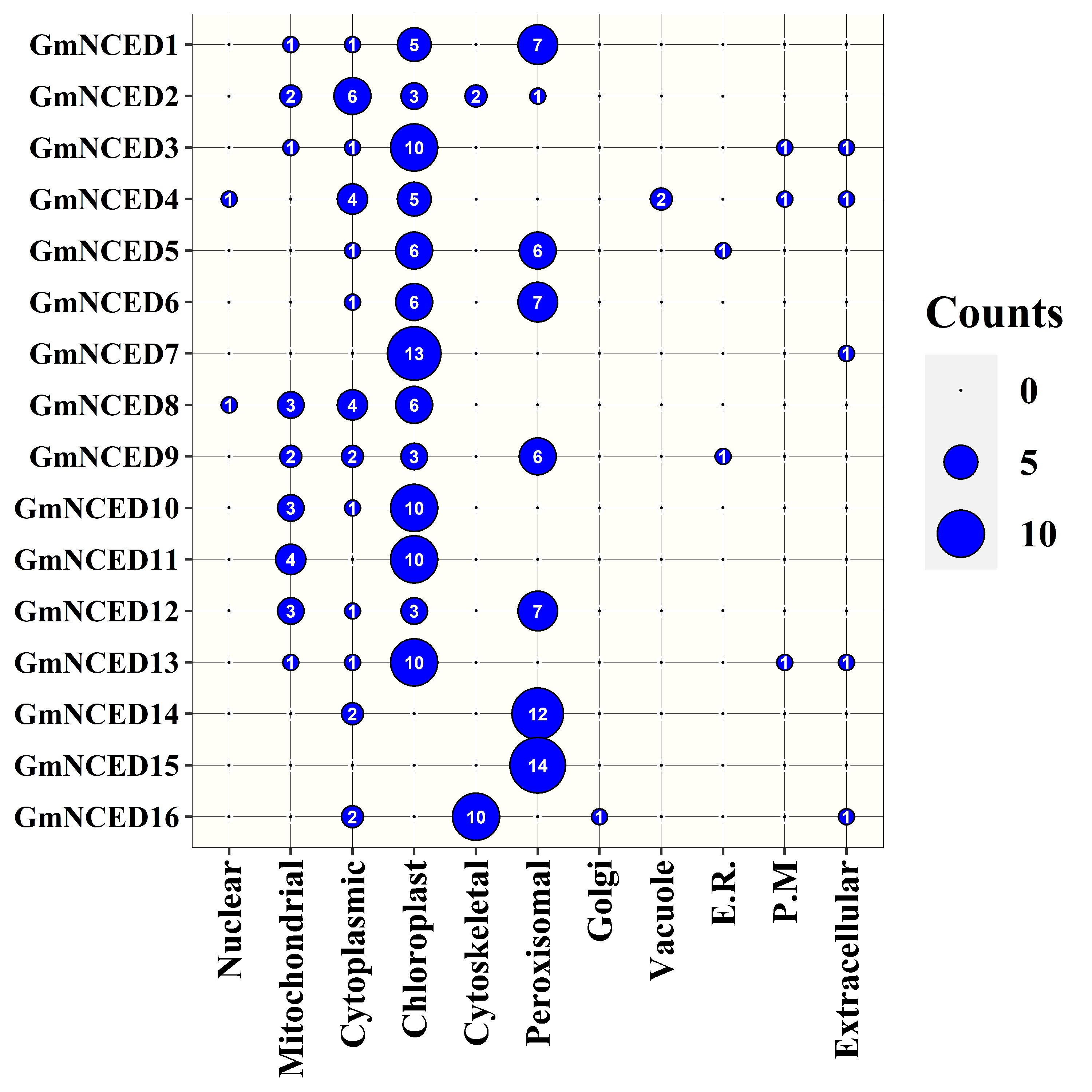

Supplement: S2 Fig — (TIF) [file pone.0319952.s017.tif]
